# The Timing of Differentiation of Adult Hippocampal Neurons Is Crucial for Spatial Memory

**DOI:** 10.1371/journal.pbio.0060246

**Published:** 2008-10-07

**Authors:** Stefano Farioli-Vecchioli, Daniele Saraulli, Marco Costanzi, Simone Pacioni, Irene Cinà, Massimiliano Aceti, Laura Micheli, Alberto Bacci, Vincenzo Cestari, Felice Tirone

**Affiliations:** 1 Institute of Neurobiology and Molecular Medicine, Consiglio Nazionale delle Ricerche, Fondazione S. Lucia, Rome, Italy; 2 Institute of Neuroscience, Consiglio Nazionale delle Ricerche, Rome, Italy; 3 LUMSA University, Faculty of Educational Science, Rome, Italy; 4 European Brain Research Institute, Rome, Italy; Baylor College of Medicine, United States of America

## Abstract

Adult neurogenesis in the dentate gyrus plays a critical role in hippocampus-dependent spatial learning. It remains unknown, however, how new neurons become functionally integrated into spatial circuits and contribute to hippocampus-mediated forms of learning and memory. To investigate these issues, we used a mouse model in which the differentiation of adult-generated dentate gyrus neurons can be anticipated by conditionally expressing the pro-differentiative gene *PC3* (*Tis21*/*BTG2*) in nestin-positive progenitor cells. In contrast to previous studies that affected the number of newly generated neurons, this strategy selectively changes their timing of differentiation. New, adult-generated dentate gyrus progenitors, in which the *PC3* transgene was expressed, showed accelerated differentiation and significantly reduced dendritic arborization and spine density. Functionally, this genetic manipulation specifically affected different hippocampus-dependent learning and memory tasks, including contextual fear conditioning, and selectively reduced synaptic plasticity in the dentate gyrus. Morphological and functional analyses of hippocampal neurons at different stages of differentiation, following transgene activation within defined time-windows, revealed that the new, adult-generated neurons up to 3–4 weeks of age are required not only to acquire new spatial information but also to use previously consolidated memories. Thus, the correct unwinding of these key memory functions, which can be an expression of the ability of adult-generated neurons to link subsequent events in memory circuits, is critically dependent on the correct timing of the initial stages of neuron maturation and connection to existing circuits.

## Author Summary

Previous studies have implicated adult-born hippocampal neurons in the formation of spatial and contextual memories by using mouse models where newly generated neurons are either eliminated or increased in number. Nonetheless, how new neurons are integrated in the existing circuits and contribute to memory formation still awaits clarification. Toward this end, we have developed a different approach, using a mouse model that accelerates the differentiation of the newly generated neurons without altering their number, and offers the possibility to induce the process at any chosen moment. We show that the new neurons pass through their early stages of maturation faster and, though establishing connections with the existing neuronal circuits, fail to function properly. In fact, mice are not only unable to learn new spatial information, but they are also unable to use previously acquired memories. These results demonstrate that the appropriate timing of maturation of new neurons is important for their adult performance in memory circuits, i.e., to integrate new memory traces and recall previous events.

## Abstract 3

The early stages of hippocampal neuron maturation are critical for establishing the connectivity to existing circuits that is required for normal spatial learning and memory.

## Introduction

Observations in mammals and birds have revealed that neurogenesis continues in the dentate gyrus of the hippocampus throughout adulthood, due to the presence of progenitor cells localized in the innermost part of the granule cell layer, the subgranular zone (SGZ, [1–3]). These progenitor cells continue to proliferate and generate new dentate granule neurons for the entire life of the organism [4,5].

The process of adult hippocampal neurogenesis originates from dividing putative neural stem cells [6] and has been tentatively divided into six developmental stages [7], in which putative neural stem cells (named type-1 cells) develop into post-mitotic neurons through three consecutive stages of progenitor cells (type-2ab and type-3 cells; [8–10]). This process is thought to govern the number and the differentiation of adult-generated neurons [11].

Newborn neurons become functionally integrated into existing dentate gyrus circuitry within 3 wk, extending their axons to CA3, as indicated by morphological and electrophysiological studies [12–20]. Interestingly, recent observations indicate that new neurons of the dentate gyrus become functionally active in learning circuits at late stages of their maturation (∼4–6 postnatal weeks, [21]).

Adult hippocampal neurogenesis is required for hippocampus-dependent learning and memory [22,23]. Indeed, the almost complete ablation of neurogenesis by an antimitotic toxin, x-ray irradiation, or virus-activated pro-drugs results in profound cognitive deficits [24–28]. On the other hand, learning and/or physical exercise can enhance neurogenesis in the dentate gyrus, suggesting a two-way relationship between the generation of new neurons in the adult hippocampus and cognitive processes [9, 29–33]. Recently, it has been hypothesized that new, adult-generated neurons confer to the dentate gyrus the basic ability to encode the timing of new memories by integrating new events on the pre-existing memory circuits [23].

The strategies used so far to reveal the functional role of adult neurogenesis deeply affected the total number of new neurons, either by severely reducing neuronal progenitors [24,26] or by increasing the number of new neurons generated [29]. Nonetheless, the ablation of new neurons by toxin or irradiation in some instance did not affect certain hippocampus-dependent tasks of spatial learning, such as water maze or contextual fear conditioning [24,26,34,35]. In this context, the role of the specific differentiation steps during neurogenesis and dynamics of integration of new neurons into existing circuitry remains unknown [36].

To address this issue, we used the approach of selectively enhancing the differentiation of progenitor cells in adult dentate gyrus. We conditionally expressed the gene *PC3* (also known as *Tis21* or *BTG2*, see [37] for review; GenBank (http://www.ncbi.nlm.nih.gov/Genbank/) accession number M60921) in nestin-positive progenitor cells (type-1 and type-2; [7]) of the adult dentate gyrus. *PC3* is normally expressed in neuronal precursors immediately before the last asymmetric division [38,39] and, during neurogenesis, it is known to induce their terminal differentiation in several areas of the CNS [40,41].

We found that nestin-driven expression of *PC3* resulted in premature differentiation of adult-generated dentate gyrus neurons, with a reduction in the number of type-1 and type-2 neuronal progenitors. This genetic manipulation did not change the overall number of newly generated neurons; rather it altered their differentiation timing and resulted in profound changes in newborn neuron morphology. Remarkably, early *PC3* expression caused a severe impairment in performance on the different hippocampus-dependent spatial learning and memory tests used and resulted in a selective reduction in synaptic plasticity in the dentate gyrus. These results indicate that early stages of adult neurogenesis are crucial for the correct functional integration of newborn neurons into cognitive hippocampal circuits.

## Results

### Adult Hippocampal Granule Precursors Are Induced to Differentiate by *PC3*

Up-regulation of *PC3* induces neural precursors to shift from proliferation to differentiation, thereby promoting the generation of new neurons [41]. Such enhancement of neurogenesis has been observed so far in neuroblasts of the neural tube and in cerebellar granule cell precursors, and follows inhibition of G1 to S phase progression and stimulation of proneural genes [41,42].

We reasoned that conditionally controlling the differentiation of progenitor cells in the adult dentate gyrus would allow a selective analysis of their involvement in spatial memory networks.

**Figure 1:**
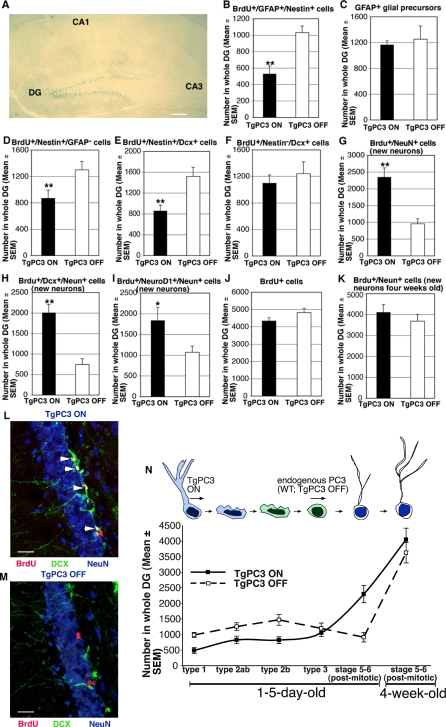
Activation of the *PC3* Transgene Accelerates the Differentiation of 1–5-d-Old Adult-Generated Dentate Gyrus Stem and Progenitor Cells (A) Selective targeting to the dentate gyrus in P95 mice of the *PC3* transgene (X-gal staining, blue) conditionally activated since P30 by doxycycline treatment (scale bar, 615 μm). New stem and progenitor cells (B–F, J) as well as new post-mitotic neurons (G–I) 1–5-d-old were detected by incorporation of BrdU after five daily injections in P95 Tg*PC3* OFF and ON mice (the latter having the transgene active), and were classified by analyzing the expression of specific markers through multiple-labeling and confocal microscopy detection. (B) The number of putative stem cells (type-1; BrdU/GFAP/nestin-positive) decreased about 50% in mice with activated *PC3* transgene. (C) This decrease was specific, given that the total number of GFAP-positive cells was only slightly reduced. (D) Activation of the *PC3* transgene also reduced a the number of new progenitor cells type-2ab (BrdU/GFAP-positive and nestin-negative), (E) type-2b (BrdU/nestin/DCX-positive), and (F) type-3 (BrdU/DCX-positive and nestin-negative). (G) Conversely, the new differentiating early post-mitotic neurons at stage 5 and 6 (BrdU/NeuN-positive), or (H) those specifically at stage 6 (BrdU/DCX/NeuN-positive), presented a strong, significant increase in mice with activated *PC3* transgene. (I) The new differentiating neurons, whose number is increased by activation of the *PC3* transgene, also expressed *NeuroD1*, which is required for their differentiation [47]. (J) Nonetheless, in mice with active *PC3* transgene, no significant change was observed in the total number of new 1–5-d-old neurons in dentate gyrus—as detected by BrdU incorporation—or (K) in the total number of new terminally differentiated neurons (4 wk old, positive to BrdU and NeuN). These findings indicate, as a whole, that activation of the *PC3* transgene selectively accelerates the transition of stem and progenitor cells toward a differentiated state. The cell numbers shown in (B–K) were measured as described in Materials and Methods and are represented as mean ± SEM from the analysis of at least three animals per group. (L and M) Representative confocal images of new 1–5-d-old dentate gyrus neurons labeled by BrdU/DCX/NeuN-positive (stage 6; red, green, blue, respectively) in Tg*PC3* ON and OFF mice. The merge shows neurons positive for all three antigens as yellow cells present in Tg*PC3* ON, indicated by white arrowheads; scale bar, 25 μm. DG, dentate gyrus. *p < 0.05 versus Tg*PC3* OFF; **p < 0.02 versus Tg*PC3* OFF; Student's *t*-test. (N) Summary from data of Figure 1 of the effect of the activation of Tg*PC3* on new progenitor cells and post-mitotic neurons.

Thus, to start, we tested whether the differentiation of adult-generated newborn dentate gyrus neurons was influenced by *PC3*, which begins to be physiologically expressed in progenitor cells before differentiation, at the moment of their final mitosis (SF-V and FT, unpublished data). Up-regulation of *PC3* was activated earlier in nestin-expressing adult hippocampal stem and progenitor cells (type-1 and type-2; [7]) of bitransgenic nestin-rtTA/TRE-*PC3* mice (hereafter named Tg*PC3*). This was achieved by conditional expression from postnatal day 30 (P30) onward of the transgene under control of the nestin promoter, through administration of doxycycline, as previously shown [41]. Sixty days after activation of the *PC3* transgene, mice received five daily injections of BrdU (at P90–P94) to detect new progenitor cells and/or neurons, immediately followed by immunohistochemical analyses. Analysis of the expression of transgenic *PC3* in P95 mice with the *PC3* transgene active (named Tg*PC3* ON mice) indicated targeting to the dentate gyrus, as visualized by X-gal staining, which revealed the β-galactosidase activity of the β*-geo* reporter gene fused to the nestin-rtTA transgene (Figure 1A).

Among the different cell populations of the dentate gyrus, the glial fibrillary acidic protein (GFAP)-expressing astroglia are considered to be dividing putative neural stem cells from which neuronal progenitor cells originate, given their ability to repopulate the dentate gyrus after cytotoxic ablation of dividing cells [6]. These stem cells are identified primarily by the expression of *GFAP*, accompanied by *nestin* or also *Sox2* [8,10,43,44] and have been defined as type-1 cells [7]. We found that, in Tg*PC3* ON mice at P95, the BrdU^+^/GFAP^+^/nestin^+^ cells—corresponding to 1–5-d-old type-1 cells—decreased significantly with respect to Tg*PC3* OFF control mice (about 40%; Figure 1B, *p* < 0.0003). The whole GFAP-positive astroglial cell population decreased only slightly, confirming the selectivity of effect on type-1 cells (Figure 1C). Since no difference between Tg*PC3* OFF and wild-type (WT) mice was found in these and in the following immunohistochemical analyses (unpublished data; *p* > 0.05), only Tg*PC3* OFF were considered as controls.

Next, we analyzed the transiently amplifying progenitor cells derived from type-1 cells. These progenitors express nestin but lack GFAP and astrocytic features and are divided into two subgroups based on the absence or presence of the immature neuronal marker, doublecortin (DCX) (named type-2a and type-2b, respectively; [8–10]). A further group of progenitor cells lacks nestin but expresses DCX (type-3; [9]). We observed that Tg*PC3* ON mice presented a significant decrease of 35% in the whole type-2a/type-2b population of 1–5-d-old newborn progenitor cells (identified as BrdU^+^/nestin^+^/GFAP^–^; *p* < 0.02, Figure 1D) and of 45% in type-2b progenitor cells (i.e., BrdU^+^/nestin^+^/DCX^+^; *p* < 0.002, Figure 1E). Moreover, Tg*PC3* ON mice also showed a reduction, albeit not significant, of type-3 progenitor cells (identified as BrdU^+^/nestin^−^/DCX^+^; Figure 1F), consistent with the fact that at this stage, the nestin promoter becomes physiologically inactive and thus ceases to drive the expression of transgenic *PC3*. A control of the expression of transgenic *PC3* in nestin-positive type-1and type-2ab progenitor cell types, as determined by β-galactosidase expression, is shown in Figure S1A–S1D.

Type-3 progenitor cells generate early post-mitotic neurons identified by expression of the neuronal-specific differentiation marker, NeuN (stage 5 and 6; [45]). In contrast to progenitor cells type-1–type-3, in Tg*PC3* ON mice the number of early post-mitotic, differentiated neurons up to 5 d old expressing NeuN increased considerably—more than twice than in control mice (identified as Brdu^+^/NeuN^+^ as well as Brdu^+^/DCX^+^/NeuN^+^; *p* < 0.0001; Figure 1G and 1H, respectively). Representative images of Brdu^+^/DCX^+^/NeuN^+^ cells are shown in Figure 1L and 1M. These new neurons, which increased in number by activation of the *PC3* transgene, were also positive for *NeuroD1* (Brdu^+^/NeuroD1^+^/NeuN^+^; Figure 1I), whose expression begins in type-2b progenitor cells, is maintained in post-mitotic hippocampal granule neurons, and is required for differentiation [46,47]. We conclude that the nestin promoter-driven expression of transgenic *PC3* accelerates the shift of adult-generated hippocampal stem and progenitors cells towards a post-mitotic, terminally differentiated phenotype, corresponding to NeuN-expressing neurons stage 5 and 6.

Notably, *PC3* transgene activation did not change the total number of 1–5-d-old new progenitors generated in the dentate gyrus (BrdU^+^ cells analyzed at P95 after completion of BrdU injections; Figure 1J) or the total number of 4-wk-old new neurons (BrdU^+^/NeuN^+^ cells analyzed at P116; Figure 1K). The effects of transgene activation on the relative abundance of new dentate gyrus progenitor cell types and neurons is summarized in Figure 1N. In addition, no significant change was detected in the number of proliferating progenitor cells of the whole dentate gyrus in mice with active transgene with respect to control mice, as detected by Ki67 labeling (Figure S2A). Furthermore, an analysis of proliferating progenitor cell types expressing Ki67 showed that in Tg*PC3* mice, the number of type-1 (Ki67^+^/nestin^+^/GFAP^+^; *p* > 0.05), type-2ab (Ki67^+^/nestin^+^/GFAP^–^; *p* < 0.01) and type-2b (Ki67^+^/nestin^+^/DCX^+^; *p* < 0.01) decreased, whereas that of type-3 progenitor cells increased significantly (Ki67^+^/nestin^–^/DCX^+^; *p* < 0.01; Figure S2B–S2F).

Together, these data indicate that *PC3* selectively accelerates the transition of stem and progenitor cells (type-1 to type-3) toward a post-mitotic differentiated state without affecting the following: (i) the total number of proliferating cells; (ii) the total number of newly generated neurons; or (iii) the final number of neurons differentiated. The decrease in the number of proliferating Ki67^+^/nestin^+^ type-1–type-2ab progenitor cells expressing *PC3* is consistent with the notion that *PC3* expression is associated to the neurogenic asymmetric type of division in neuroblasts [37–39].

To test whether activation of the *PC3* transgene caused nonspecific changes, a stereological analysis of the hippocampus was conducted. Activation of the *PC3* transgene after P30 did not alter the volume of the dentate gyrus or the whole hippocampus (Figure S3A and S3B) or the total cell number in the dentate gyrus (Figure S3C). Moreover, there was no evidence of reduced cell survival either in the whole hippocampus, as defined by TUNEL analysis (Figure S3D), or within different subpopulations, as defined by labeling with the apoptotic marker caspase-3 [48] (Figure S3E–S3J). Also, no evident alteration in distribution of mature granule neurons or in morphology was detected in the hippocampus or in the whole brain (unpublished data).

In conclusion, these results show that the activation of the *PC3* transgene accelerates the process of differentiation of dentate gyrus progenitor cells, thus providing a tool to specifically analyze the relationship between timing of hippocampal neurogenesis and spatial learning.

We wished also to verify whether the expression of *PC3* had any effect on nestin-positive neural cells in the other adult neurogenic niche, i.e., the subventricular zone (SVZ), comprising type B astrocytic-like progenitors, type C transit amplifying cells, and type A migrating neuroblasts [49–51]. We observed that the number of type B and type C cells, labeled by BrdU/GFAP and BrdU/NG2, respectively, did not change significantly in Tg*PC3* ON mice (*p* > 0.05; Figure S4A and S4B); on the other hand, the number of type A cells, corresponding to immature neurons derived from type C cells and identified by BrdU/DCX staining, was significantly lower than in control mice (40% decrease, *p* < 0.01; Figure S4C). Conversely, differentiated SVZ neurons up to 5 d old (BrdU^+^/NeuN^+^ cells) increased more than 2-fold in Tg*PC3* ON mice (*p* < 0.01; Figure S4D), although the final number of new neurons generated in SVZ, as analyzed measuring BrdU^+^/NeuN^+^ cells 3 wk later (at P116) in their final migratory destination, i.e., the olfactory bulb, did not differ in Tg*PC3* ON and OFF mice (unpublished data).

This suggests that *PC3* accelerated the differentiation of SVZ cells, as observed for the cells of the dentate gyrus. It should be emphasized, however, that SVZ cells are not involved in spatial memory processes, being anatomically and functionally independent from those of the dentate gyrus, since they migrate through the rostral migratory stream to the olfactory bulb where they play a specific role in the olfactory processes [49,52].

### Reduced Dendritic Arborization and Spine Density in Adult-Generated Neurons in the Dentate Gyrus Prematurely Differentiated

We then investigated whether early-differentiated dentate gyrus neurons in Tg*PC3* ON mice showed an altered morphology in the three different stages of the morphogenetic process underlying the maturation of granule neurons from progenitors in the SGZ of dentate gyrus. We labeled the newly generated granule neurons in adult hippocampus of Tg*PC3* mice by infecting the dentate gyrus region with a retrovirus produced by the vector CAG-GFP [19]. Highly concentrated retrovirus (about 10^8^ pfu/ml) was delivered through sterotaxic surgery in the dentate gyrus of Tg*PC3* mice at P95, two months after activation of the transgene, according to the time schedule followed for immunohistochemical analyses. CAG-GFP vector is replication incompetent; thus, only dividing cells at the moment of surgery could be infected.

**Figure 2:**
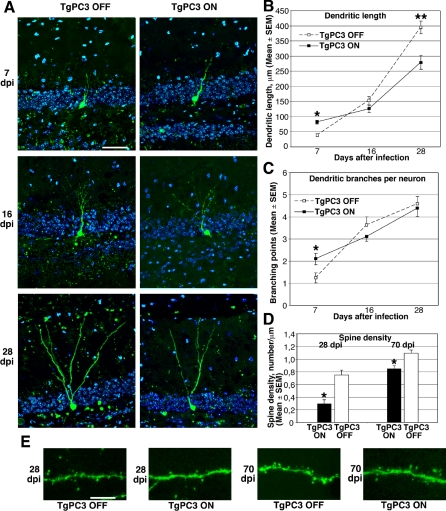
Morphological Analysis of the Development of Adult-Generated Dentate Gyrus Neurons (A) Representative morphology of adult-generated neurons labeled with GFP by retrovirus-mediated gene transduction, analyzed from 7–28 dpi, in control (Tg*PC3* OFF) or activated transgenic mice (Tg*PC3* ON; the transgene was activated 60 d before infection, according to the time schedule of Figure 1). The GFP^+^ neurons (green) are localized at the hilar border of the dentate gyrus, whose cells are identified by Hoechst 33258 staining (blue). The molecular layer is localized at the upper side of the images. Scale bar, 50 μm. (B) Quantification of the dendritic length of adult-generated GFP^+^ neurons at 7, 16, and 28 dpi. (*F*(5, 148) = 73.7; *p* < 0.0001, ANOVA. *p < 0.05 or **p < 0.001 between Tg*PC3* ON and OFF groups at the same day post infection, ANOVA Fisher's PLSD post-hoc analysis). (C) Quantification of branching points at 7, 16, and 28 dpi. (*F*(5, 148) = 19.8; *p* < 0.0001, ANOVA. *p < 0.05 between 7 dpi Tg*PC3* ON and OFF groups, ANOVA Fisher's PLSD post-hoc analysis). (D) Quantification of spine density in the dendritic processes of GFP^+^ neurons analyzed at 28 dpi and 70 dpi. *p < 0.002, Student's *t*-test. Data in (B–D) are presented as mean ± SEM; at least three animals per group were analyzed. (E) Representative confocal images of dendrites and spines in 28 dpi and 70 dpi GFP^+^ dentate gyrus neurons. Scale bar, 12 μm.

The soma of the majority of GFP^+^ neurons detected in control mice was localized on the hilar border of the granule cell layer of the dentate gyrus at the different time points analyzed, i.e., throughout 7 −28 days post-infection (dpi). The most evident developmental feature of GFP^+^ neurons, detected at different dpi in Tg*PC3* mice with either active or inactive transgene, was the progressive growth of dendrites, whose lengths, shorter than the width of the dentate gyrus blade at 7 dpi, increased greatly at 16 dpi and further at 28 dpi, reaching far outside of the dentate gyrus. During these last two time points, the dendritic growth was accompanied by an increase in the complexity of arborization (Figure 2A). There were, however, clear differences in neuronal morphology between Tg*PC3* ON and Tg*PC3* OFF mice, which we evaluated by scoring dendritic length and branching points. Analysis of GFP^+^ neurons at 7 dpi revealed a significantly increased dendritic length in Tg*PC3* ON mice compared with Tg*PC3* OFF. This difference, however, was reversed in GFP^+^ neurons at 16 dpi and more clearly at 28 dpi, when the dendritic lengths in Tg*PC3* ON mice were significantly shorter than those in Tg*PC3* OFF mice (Figure 2B). Similarly, the number of branching points in GFP^+^ neurons at 7 dpi was significantly greater in Tg*PC3* ON than in Tg*PC3* OFF mice, but again this difference was transient, because in GFP^+^ neurons at 16 and 28 dpi, the number of branching points became equivalent in the two groups (Figure 2C).

To further evaluate the dendritic complexity, we analyzed the density of spines. The dendritic spine is the locus of connection to excitatory synaptic input, mainly glutamatergic [53]; therefore, the process of spine formation has consequences in the functionality of neurons. Normally, spine growth starts in the adult-generated dentate gyrus neurons at about 16 dpi and reaches a plateau at 56 dpi, but at 28 dpi the exponential phase of growth has already ceased, and this stage can be considered representative of the attainment of the morphological maturity of the neuron [19]. Therefore, we focused our analyses at 28 dpi, and also at 70 dpi, when the plateau of growth is attained. Quantification of spine density in dentate gyrus neurons indicated a significant reduction both at 28 dpi and at 70 dpi in Tg*PC3* mice with active transgene, compared to mice with inactive transgene (Figure 2D). However, at 28 dpi the decrease of spine density was substantial (60%), whereas at 70 dpi, it was mild (15% decrease; Figure 2D and 2E).

We conclude that the dendritic length of new dentate gyrus neurons in Tg*PC3* ON mice, albeit greater than in control mice at 7 dpi, appeared significantly shorter at 28 dpi, associated with reduced spine density. This evidence points to a decrease in dendritic growth and in spine number during the developmental stages between 7 and 28 dpi; a partial recovery of normal values of spine density may slowly occur during the following stages. The absence of alteration in the branching points would indicate that no major morphological alterations occur, other than those likely consequent to a faster attainment of the end point of the maturation process.

### Tg*PC3* ON Mice Are Severely Impaired in Spatial Learning and Memory Tasks

**Figure 3:**
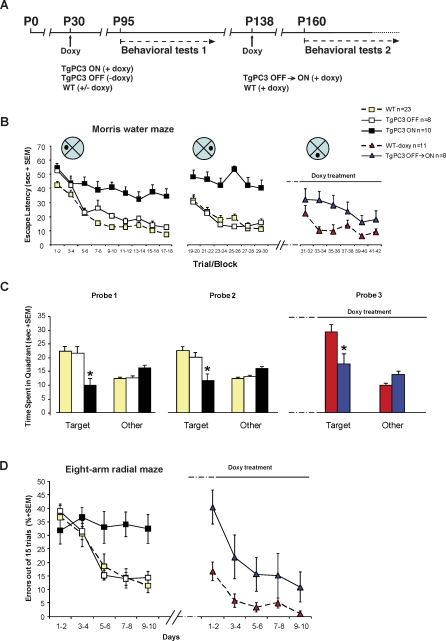
*PC3* Transgene Activation Impairs Learning in the Morris Water Maze and Radial Maze (A) Experimental timeline as a function of mouse age in days. P95 mice were subjected to a first session of spatial learning and memory tests (behavioral tests 1; Tg*PC3* ON mice had the transgene active starting from P30 throughout the experiment). At the end of the first session, Tg*PC3* OFF mice were treated with doxycycline to activate the *PC3* transgene and subjected to a further experimental session (behavioral tests 2). Arrows indicate the starting days of doxycycline treatment. (B) Tg*PC3* ON (*n* = 10), Tg*PC3* OFF (*n* = 8) and WT (*n* = 23) mice were trained in the Morris water maze for 18 trials (six trials per day; trial/block 1–18), followed by 2 d of reversal learning (trial/block 19–30) in which the platform was moved to the opposite position. After the *PC3* transgene activation, Tg*PC3* OFF (labelled as Tg*PC3* OFF → ON, *n* = 8) and WT-doxy (*n* = 11) mice were subjected to a new learning session (trial/block 31–42) in which the platform was moved to a novel position in the pool. Escape latency is expressed in seconds required to reach the platform. The performance of Tg*PC3* ON mice was significantly impaired during both learning (left) and reversal learning (middle) in comparison with both Tg*PC3* OFF and WT. Tg*PC3* OFF → ON mice were significantly impaired in the new learning (right) in comparison with WT-doxy. (C) Probe trials expressed as time (s) spent in the target quadrant (Target) or in the control quadrants (Other). Compared to Tg*PC3* OFF and WT, Tg*PC3* ON mice spent a significantly smaller amount of time in the target quadrant both in probe 1 (left) and probe 2 (middle). During probe 3 (right), Tg*PC3* OFF → ON mice spent a significantly smaller amount of time in the target quadrant compared to WT-doxy. (D) Mice were trained for 10 d in a fully baited, eight-arm radial maze. The learning performance is expressed as percentage of errors until eight correct arm choices have been observed or after the maximal permitted number of trials. The performance of Tg*PC3* ON mice (*n* = 10) was significantly impaired in comparison with those of Tg*PC3* OFF (*n* = 8) and WT (*n* = 23) (left). In session 2 of the behavioral tests, the performance of Tg*PC3* OFF → ON mice (*n* = 8) was significantly impaired in comparison with that of WT-doxy (*n* = 11) (right). *p < 0.05 in comparison with all other groups.

Spatial learning was tested in the Morris water maze [54,55]. In this task, which is mainly dependent on an intact hippocampus [54,56], mice learn across daily sessions to find a hidden escape platform using extra-maze visual cues. A first session of experiments, carried out in 3-mo-old WT, Tg*PC3* OFF and Tg*PC3* ON mice (whose transgene had been activated at P30; see time-schedule in Figure 3A), showed significant differences among groups in escape latencies both in learning (trials 1–18; *F*(2,38) = 32.04; *p* < 0.0001) and reversal learning (trials 19–30; *F*(2,38) = 34.15; *p* < 0.0001). As indicated by post-hoc comparisons (Duncan multiple range test), Tg*PC3* ON mice were dramatically impaired (*p* < 0.05) both in learning and reversal learning compared to WT and Tg*PC3* OFF mice, whereas no significant differences were found between the performances of Tg*PC3* OFF and WT mice (Figure 3B). Moreover, both in the first (*p* < 0.001) and the second (*p* < 0.01) probe trial, carried out 24 h after learning and reversal learning, respectively, Tg*PC3* ON mice spent a significantly smaller amount of time in the target quadrant, compared to both Tg*PC3* OFF and WT mice. No significant differences were found between Tg*PC3* OFF and WT mice (Figure 3C). In this analysis, we considered WT mice, either doxy-treated or doxy-free, as a single control sample, because no significant differences were found between the two groups. An analysis of behavioral aspects not related to learning, i.e., wall hugging behavior (thigmotaxys) and swimming speed, did not show significant differences between Tg*PC3* ON, Tg*PC3* OFF, and WT mice either during the first or the second behavioral session (Figure S5A and S5B). However, increased levels of thigmotaxys were detectable at later stages of training in Tg*PC3* ON mice, which may reflect their poor learning.

To further test the effect of the *PC3* transgene activation on spatial memory, we submitted transgenic and control mice to a less stressful behavioral task compared to the water maze, namely the eight-arm radial maze [57,58]. In this task, mice have to search for a food pellet located at the end of each arm. Reentering a previously visited arm was considered as an error. Significant differences in the percentage of errors were found among groups (*F*(2,38) = 6.71; *p* < 0.005). As shown by post-hoc comparisons (Duncan multiple range test), the performance of TgPC3 ON mice was severely impaired (*p* < 0.05) in comparison to both Tg*PC3* OFF and WT mice, whereas no significant differences were found between these latter groups (Figure 3D, left).

Given that the above behavioral tests were conducted in Tg*PC3* ON mice in the presence of an active transgene, we wished to verify whether the observed behavioral phenotype could be related to indirect effects of *PC3* over-expression in nestin-positive cells on neighboring, mature granule cells. Thus, we tested spatial learning in the Morris water maze using Tg*PC3* ON mice in which the transgene was activated at P30 and inactivated at P95, i.e., prior to behavioral tests. To this aim, doxycycline treatment was suspended at P90, to allow 5 d of metabolization of the residual levels (Figure S6A). The performance of Tg*PC3* ON mice was significantly impaired in learning and reversal learning, as well as in the first and in the second probe trial, compared to WT mice (Figure S6B and S6C). Thus, we can exclude indirect cell-mediated effects by nestin-positive cells expressing the *PC3* transgene during the behavioral tests.

Altogether, these behavioral results indicate that, in *PC3* transgenic mice, the accelerated differentiation of dentate gyrus adult progenitors dramatically impairs learning and memory of spatial information.

### Subsequent *PC3* Gene Activation in Trained Mice Results in Spatial Learning and Memory Deficits

To verify whether genetically altered neurogenesis could impair learning performance in previously experienced tasks, Tg*PC3* OFF mice and their controls were treated with doxycycline (doxy; Tg*PC3* OFF → ON and WT-doxy, respectively) following the first experimental phase. In addition, for this set of experiments, the gene was activated for a shorter period of time (see time schedule in Figure 3A). During treatment, both Tg*PC3* OFF → ON and WT-doxy mice were submitted to a second session of spatial learning and memory tasks. A new piece of information was introduced in the Morris water maze by moving the hidden platform to a different location. By contrast, in the radial maze, all mice were tested with the same procedure used in the first experimental phase. This differential approach was used to test for the ability of the mice to use previously acquired information.

In the Morris water maze, Tg*PC3* OFF → ON mice were impaired in learning the novel position of the platform in comparison to controls (Figure 3B, trials 31–42; *F*(1,17) = 5.25; *p* < 0.05). In the probe test, Tg*PC3* OFF → ON mice spent a significantly smaller amount of time in the target quadrant in comparison to WT-doxy mice (Figure 3C, probe 3; *p* < 0.05). Similarly, in the radial maze, Tg*PC3* OFF → ON mice committed a significantly greater number of errors compared to WT-doxy mice (Figure 3D, right; *F*(1,17) = 7.79; *p* < 0.05).

These results indicate that the anticipated differentiation of dentate gyrus neural progenitors produces a remarkable impairment in tasks properly solved by transgenic mice before the *PC3* transgene activation. Thus, the normal differentiation timing of dentate gyrus neural precursors emerges as a major constraint to learning performance, even in a previously experienced spatial setting, either in the presence or absence of new information.

### Tg*PC3* ON Mice Are Impaired in the Contextual, but Not in the Cued, Version of Fear Conditioning

**Figure 4:**
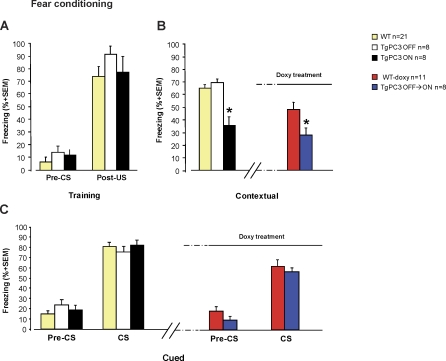
*PC3* Transgene Activation Impairs Contextual but Not Cued Fear Conditioning (A) Percentage of time spent in freezing behavior by Tg*PC3* ON (*n* = 8), Tg*PC3* OFF (*n* = 8), and WT (*n* = 21) mice during training in the conditioning box. Training consisted of an acclimatizing period (Pre-CS, 120 s) followed by a tone presentation (CS, 30 s) paired, in the last 2 s, with a foot-shock (US). After CS-US pairing, the animals were left in the conditioning box for a further 30 s (Post-US). During training, no significant differences in the level of freezing were observed among the different groups of mice. (B) Percentage of time spent in freezing behavior in the contextual test, carried out 24 h after training, by Tg*PC3* ON, Tg*PC3* OFF, and WT mice. Tg*PC3* ON mice showed a significant reduction in the level of freezing compared with Tg*PC3* OFF and WT (left). In session 2 of the behavioral tests, Tg*PC3* OFF → ON mice showed a significant memory impairment compared with WT-doxy (right). (C) Percentage of time spent in freezing behavior in the cued test, carried out 2 h after the contextual test in a different setting, by Tg*PC3* ON, Tg*PC3* OFF, and WT mice. No significant differences in the retention level were observed among the different groups of mice during either Pre-CS or CS phases (left). In session 2 of the behavioral tests, no significant differences in the level of freezing were observed between Tg*PC3* OFF → ON and WT-doxy mice (right). *p < 0.05 in comparison with all other groups.

We then investigated the effects on memory of the *PC3* transgene activation in a fear-related learning task, namely contextual and cued fear conditioning, involving mainly the hippocampus and amygdala, respectively [59,60]. In this task, immobility (freezing), a natural reaction elicited in mice by aversive stimuli, was recorded and considered a measure of fear memory. Animals were preliminarily trained in the conditioning box (chamber A), in which a conditioned stimulus (CS) and unconditioned stimulus (US; see Materials and Methods) were paired. During the training, no significant effect of the genotype was observed among groups in the level of freezing, both in the pre-CS (*F*(2,34) = 1.47; *p* = not significant) and the post-US (*F*(2,34) = 0.91; *p* = not significant) phases, and all the groups of mice reacted alike to the US (*F*(1,34) = 115.52; *p* = 0.0001; Figure 4A). In the contextual test, analysis of the percentage of freezing showed significant differences between groups (*F*(2,34) = 17.68; *p* < 0.0001). As shown by post-hoc comparisons (Duncan multiple range test), Tg*PC3* ON mice spent a significantly smaller amount of time (*p* < 0.0001) in freezing behavior compared to both Tg*PC3* OFF and WT mice, whereas no significant differences were found between Tg*PC3* OFF and WT mice (Figure 4B, left). Two hours after the contextual test, cued memory was assessed by the administration of the CS in a different setting (chamber B). No significant differences between groups emerged (*F*(2,34) = 0.15; *p* = not significant), whereas a significant effect of the CS was observed (*F*(1,34) = 318.04; *p* < 0.0001), that is to say that, in the novel environment, all groups of mice exhibited an appreciable level of freezing only during the CS administration (Figure 4C, left).

### Transgene Activation in TgPC3 OFF Mice Induces a Significant Contextual Fear Memory Deficit

In the following experimental phase, Tg*PC3* OFF → ON and WT-doxy mice were submitted to a second session of fear conditioning, with an unvaried procedure (i.e., training in the same box used in the first experimental phase, followed by contextual and cued tests; see also Materials and Methods). Transgene activation significantly impaired the contextual memory (Figure 4B, right; *F*(1,17) = 6.53; *p* < 0.05), whereas no significant differences between transgenic and control mice were observed in the cued conditioning test (Figure 4C, right; *F*(1,17) = 2.72; *p* = not significant).

As in spatial tasks, these results further indicate that, also in a fear-related task, genetically altered neurogenesis produces a memory impairment for the contextual features mice had already experienced. Both in the first and the second behavioral session, the deficit was selective for memory contents processed mainly by the hippocampus (i.e., those related to spatial information), sparing the components of the experience whose processing appears to be peculiar to the amygdala.

### Premature Differentiation of Adult-Generated Granule Cell Neurons Decreases Long-Term Potentiation of Synaptic Transmission in the Dentate Gyrus Selectively

**Figure 5:**
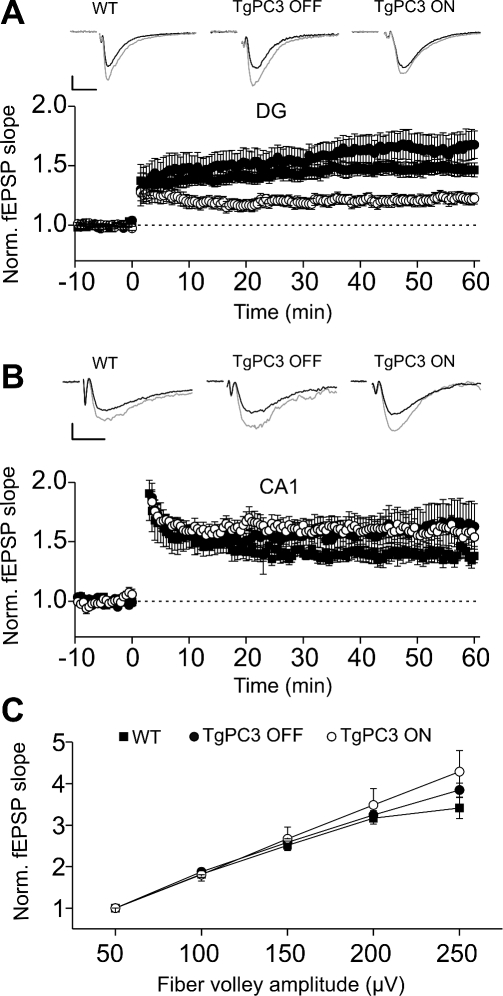
Effect of Premature Differentiation of Adult-Generated Granule Cells on Hippocampal Synaptic Plasticity (A) LTP of lateral perforant pathway synapses in the dentate gyrus is reduced in Tg*PC3* ON but not in Tg*PC3* OFF mice. Upper panel shows traces of fEPSPs immediately before (black traces) and after 50 min (gray traces) of high-frequency trains in WT, Tg*PC3* ON, and OFF mice. Averages of 10 traces for each example are shown. The stimulus artifact was digitally removed for display purposes. Scales: 5 ms and 100 μV wt; 25 μV Tg*PC3* OFF; 60 μV Tg*PC3* ON. (B) LTP of Schaffer collateral synapses measured in CA1 was unaffected by early *PC3* gene activation (*p* > 0.2). Upper panel: representative fEPSPs before (black traces) and after (gray traces) high frequency stimulations in all conditions. Shown are averages of 10 traces each. The stimulus artifact was digitally removed for display purposes. Scales: 5 ms and 60 μV wt; 100 μV Tg*PC3* OFF; 100 μV Tg*PC3* ON. (C) The slope of input-output (I/O) curves at perforant-path synapses in dentate gyrus was similar in all three conditions.

Long-term changes in synaptic strength have been described in several brain areas. In particular, hippocampal long-term potentiation (LTP) of glutamatergic synaptic transmission is believed to be the synaptic correlate of several forms of learning and memory (for reviews, see [61,62]). To test whether the above-described deficits in memory tasks caused by early expression of the *PC3* gene correlated with a change in long-term synaptic transmission, we recorded field excitatory postsynaptic potentials (fEPSPs) in the outer molecular layer of the dentate gyrus while stimulating perforant path afferents in WT, Tg*PC3* OFF, and Tg*PC3* ON mice. Robust LTP of excitatory synaptic transmission could be evoked by high-frequency stimulations (HFS: four trains of 100 stimuli at 100 Hz) in WT animals. Overall, fEPSP slopes were 1.5 ± 0.04 larger when measured 50 min after LTP induction and compared with baseline levels (*n* = 13 slices, 7 mice; *p* < 0.001; Figure 5A). Similar levels of synaptic potentiation were observed in Tg*PC3* OFF animals (normalized fEPSP slope at 50 min after HFS = 1.62 ± 0.12, *n* = 9 slices, 7 mice, *p* > 0.2 when compared with WT, Figure 5A). In Tg*PC3* ON mice, HFS evoked LTP consistently (normalized fEPSP slope was 1.2 ± 0.04, *n* = 12 slices, 7 mice, *p* < 0.001), but we found that the level of potentiation was significantly smaller when compared to WT and Tg*PC3* OFF mice (*p* < 0.001; Figure 5A). This effect on LTP was not due to overall changes in synaptic transmission, as input-output curves (fEPSP slopes versus increasing afferent fiber volley amplitudes) were similar in WT, Tg*PC3* OFF, and Tg*PC3* ON mice (Figure 5C).

Hippocampal adult neurogenesis is selectively localized in the dentate gyrus. We thus examined whether LTP deficits recorded in the Tg*PC3* ON mice were selective for dentate gyrus synapses. We then recorded fEPSPs in the CA1 area of the hippocampus while stimulating the Schaffer collateral fibers in stratum radiatum. A single train of HFS induced robust LTP in WT (*n* = 7 slices, 2 mice), Tg*PC3* OFF (*n* = 8 slices, 2 mice), and Tg*PC3* ON mice (*n* = 5 slices, 2 mice) (Figure 5B). No significant differences were observed between all three conditions tested (*p* > 0.3 in all cases).

As a whole, these results indicate that the accelerated differentiation by early *PC3* expression during adult neurogenesis affects synaptic plasticity selectively in the dentate gyrus, without altering CA3–CA1 hippocampal synapses.

### Premature Differentiation of Adult-Generated Granule Cell Neurons Reduces Their Incorporation into Spatial Memory Networks of Dentate Gyrus

**Figure 6:**
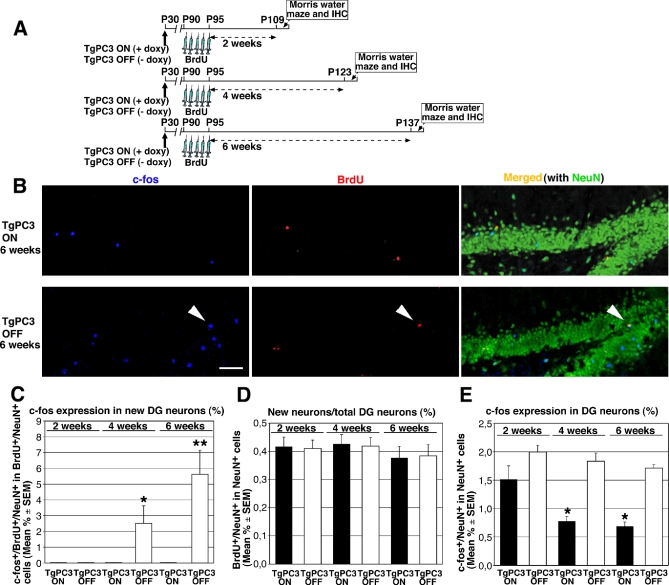
Lack of Integration into Dentate Gyrus Memory Circuits of New Neurons Derived from Dentate Gyrus Progenitor Cells Whose Differentiation Was Anticipated New neurons derived from dentate gyrus progenitor cells whose differentiation was anticipated by the *PC3* transgene do not become integrated into dentate gyrus spatial learning circuits. (A) Experimental design. 2, 4, and 6 wk after treatment with BrdU, Tg*PC3* ON/OFF and WT mice underwent Morris water maze behavioral training, which is expected to activate new neurons. (B) Representative confocal images of c-fos^+^ (blue), Brdu^+^ (red), NeuN^+^ (green; merged with c-fos^+^ and Brdu^+^) and of c-fos^+^/BrdU^+^/NeuN^+^ cells (white arrowhead) in the dentate gyrus following behavioral training in the 6-wk groups of Tg*PC3* ON and Tg*PC3* OFF mice (scale bar, 58 μm). (C) New activated neurons, identified by positivity to c-fos (c-fos^+^/BrdU^+^/NeuN^+^ cells), were quantified as percentage ratio to the number of new neurons (Brdu^+^/NeuN^+^). The fraction of new neurons activated by behavioral training was null in Tg*PC3* OFF mice undergoing behavioral training 2 weeks after treatment with BrdU (*n* = 3) but became significantly detectable 4 and 6 wk after BrdU treatment (*n* = 6 at both time points; *F*(5, 274) = 7.4; *p* < 0.0001, ANOVA. *p < 0.05 or **p < 0.02 versus the other groups, ANOVA Fisher's PLSD post-hoc analysis). By contrast, no activation of new neurons was observed in the 2-, 4-, and 6-wk groups of Tg*PC3* ON mice (*n* = 3, 6, and 6, respectively). (D) New neurons were generated with equivalent frequency in the Tg*PC3* ON and Tg*PC3* OFF groups, as indicated by the similar percentage ratio between number of new neurons (BrdU^+^/NeuN^+^) and total number of neurons (NeuN^+^ cells) identified 2, 4, and 6 wk after BrdU treatment (*F*(5, 122) = 0.24; *p* > 0.05, ANOVA). (E) After behavioral training, the presence of activated neurons in the whole neuronal population of the dentate gyrus—measured as percentage ratio of c-fos^+^/NeuN^+^ to NeuN^+^ cells—was similar in all groups of Tg*PC3* OFF mice, whereas in Tg*PC3* ON mice the presence of activated neurons was significantly reduced in the 4- and 6-wk groups, indicating a progressive decrease in the population of active neurons (*F*(5, 112) = 16.0; *p* < 0.0001, ANOVA. *p < 0.02 versus 2-, 4-, 6-wk Tg*PC3* OFF and 2-wk Tg*PC3* ON mice groups, ANOVA Fisher's PLSD post-hoc analysis). The percentage ratios shown in (C–E) were calculated from absolute cell numbers measured as described in Materials and Methods and are represented as mean ± SEM.

The decrease of LTP evoked in the dentate gyrus of mice with an activated transgene prompted us to ask whether this decrease was correlated with the function of new neurons in memory circuits. It has been shown that newly generated neurons of the dentate gyrus are progressively integrated into spatial memory-related circuits after 4–6 wk of age. Such evidence was obtained by measuring c-*fos* expression, whose activation specifically occurs in dentate gyrus neurons of mice that have undergone spatial memory training and is thus correlated with the recruitment of new neurons into spatial memory circuits [21]. Thus, we wished to define whether the anticipated differentiation of new granule neurons of dentate gyrus had an effect on the time at which they became active in memory networks and on the extent of their activation. To this end, we measured the number of new neurons at 2, 4, and 6 wk of age that become activated by a spatial memory test. Mice, either control (Tg*PC3* OFF) or with the *PC3* transgene activated 60 d before (Tg*PC3* ON), were treated with five daily injections of BrdU and then trained in different groups in the Morris water maze test 2, 4, and 6 wk later (Figure 6A). Mice were killed 1.5 h following the tests, and the number of new neurons, identified by positivity to BrdU and NeuN, was compared to the number of new neurons integrated into memory circuits, identified by c-*fos*, BrdU, and NeuN concomitant expression (Figure 6B). In Tg*PC3* OFF mice, the fraction of new neurons expressing c-*fos* was null 2 wk after birth (i.e., after BrdU injections) but reached about 2% after 4 wk and increased to about 5% after 6 wk (Figure 6C). These data are in agreement with those obtained by Frankland's group, and they indicate a progressive functional activation of new neurons within spatial circuits, correlated to their age and maturation [21]. In contrast, in mice with the *PC3* transgene activated, virtually no 2-, 4-, or 6-wk-old new neurons expressing c-*fos* were detectable, clearly indicating that new neurons did not undergo activation by spatial training (Figure 6C). The lack of induction of c-*fos* expression by spatial training in 4- and 6-wk-old neurons of Tg*PC3* ON mice corresponded to a significant learning deficit of these mice in the Morris water maze task (Figure S7A and S7B).

Moreover, in the dentate gyrus, the ratio between the total number of new neurons generated (i.e., BrdU^+^/NeuN^+^ cells) and the total number of neurons (NeuN^+^ cells) did not significantly differ in the same Tg*PC3* ON and Tg*PC3* OFF mice groups analyzed 2, 4, and 6 wk after BrdU injection (Figure 6D). Also the absolute numbers of BrdU^+^/NeuN^+^ cells in the dentate gyrus of the 2-, 4-, and 6-wk groups were equivalent in the Tg*PC3* OFF and Tg*PC3* ON mice (unpublished data). These results are consistent with data shown above (Figure 1K) and conclusively indicate that the lack of activated new neurons observed in Tg*PC3* ON mice (Figure 6C) is a genuine effect, not caused by impaired generation of new neurons.

It is notable that c-*fos* was expressed in the neuronal population of the dentate gyrus to a similar extent in all groups of control mice, as measured by the ratio between total number of activated neurons (c-*fos* ^+^/NeuN^+^ cells) and total number of neurons (NeuN^+^ cells), indicating the existence of a basal level of activation in the neuronal population. In contrast, in Tg*PC3* ON mice, the ratio between activated and total number of neurons was progressively reduced in the 2-, 4-, and 6-wk groups, with a significant difference in the 4- and 6-wk groups (Figure 6E). One possibility suggested by this finding is that the failure to generate new activated neurons in dentate gyrus in response to spatial behavioral training, observed above (Figure 6C), leads to a progressive reduction in the total number of activated neurons.

## Discussion

### Anticipated Maturation of Adult-Generated Hippocampal Progenitor Cells

The transgenic conditional model used in this study offers the possibility of analyzing the hippocampus-dependent learning and memory processes after a selective enhancement of the maturation rate of new adult-generated hippocampal neurons. In contrast, previous studies eliminated new progenitor cells and neurons from the adult hippocampus, using either antimitotic toxins, x-ray irradiation or virus-activated pro-drugs [24,26,28]. Our analysis of the dentate gyrus showed that activation of the *PC3* transgene driven by nestin promoter led to a great increase in fully differentiated, 1–5-d-old, adult-generated hippocampal granule neurons (stage 6; positive for NeuN and BrdU), accompanied by a parallel decrease in stem cells and putative transiently amplifying progenitor cells (type-1 and −2) of the same age. These results clearly indicate an accelerated transition toward terminal differentiation of the newborn progenitor cells expressing the PC3 transgene. Moreover, the analysis of fully differentiated hippocampal granule neurons of about 4 wk of age showed that the final number of neurons produced is the same in both Tg*PC3* ON and control mice. As a whole, these findings indicate that the rate of maturation of new neurons is accelerated by *PC3*, without any effect on the rate of neurogenesis—i.e., the birth rate of new neurons—and thus on the total number of new neurons generated.

Such *PC3*-dependent anticipated differentiation of progenitor cells is in line with the previously observed, intrinsic differentiative properties of *PC3* on neural precursors [41] and might result from the prevalence, in progenitor cells type-1 and type-2, of the asymmetric neurogenic type of division, with which the expression of *PC3* is associated [37–39,63].

### Deficits in Spatial Learning and Memory Are Associated with Reduced Functionality of New Neurons

The enhanced rate of maturation of the whole population of progenitor cells of the adult dentate gyrus of Tg*PC3* ON mice was clearly associated with a severe impairment in hippocampus-dependent learning and memory, as indicated by the performance of transgenic mice in different hippocampus-related behavioral tasks. In fact, learning and memory deficits were observed both in the Morris water maze and in the radial maze test. Furthermore, a significant memory deficit was also observed in the contextual fear conditioning test, in which both the hippocampus and the amygdala mediate the association between context and the aversive stimulus [59]. Conversely, no significant effect was observed in the cued version of the task, mainly characterized by a greater involvement of the amygdala formation (reviewed in [64–66]).

Such selective impairment of hippocampal spatial learning raises questions about the role played by functional and morphological modifications occurring in newborn neurons of our mouse model.

A relevant functional change observed in mice with maturation of progenitor cells enhanced by *PC3* is the decrease of LTP evoked in the dentate gyrus, an effect that appears to be specific, because no alteration of synaptic plasticity in the CA1 region is seen. It is widely believed that LTP and depression of synaptic transmission underlie several forms of learning and memory [61,62], and are often accompanied by structural changes of dendritic spines (reviewed in [67–69]). The earliest form of excitability is shown by nestin^+^ non-radial precursor cells (type-2 cells; [18]), and LTP begins to be observed in adult-generated, young (1–3-wk-old) hippocampal neurons, where it is elicited more easily than in mature, post-mitotic dentate gyrus neurons [13,16]. Thus, the reduced LTP that we observe should not depend on the reduced number of progenitor cells type-2 and type-3 present in mice with activated transgene, because they are too immature to generate LTP. Rather, the reduction of LTP may depend on new neurons that are ≥2 wks old, whose numbers are unchanged in our mouse model but whose dendritic arborization and spine density are markedly decreased. Thus, bypassing the early stages of differentiation during adult neurogenesis in neurons generated following transgene activation strongly affects the ability of dentate gyrus circuits to integrate synaptic transmission. This is further suggested by the observation that basic synaptic transmission is unchanged, as input-output curve analysis shows.

A second functional change that we observe in the dentate gyrus of transgenic mice is the lack of activation of new, fully differentiated neurons, 4–6 wk of age, in response to learning, as indicated by the absence of induction of c-*fos* expression after training. Indeed, the activation of adult-generated dentate gyrus neurons in spatial memory circuits has been linked to the induction of c-*fos* expression [21,70]. This expression is elicited in adult-generated dentate gyrus neurons, 4–6 wk of age, in mice trained in the Morris water maze, indicating that at this developmental stage the dentate gyrus neuron develops functions critical for its activation and recruitment into spatial memory circuits [21]. Hence, the absence of c-*fos* activation in 4–6-wk-old neurons that have expressed *PC3* at birth suggests that these neurons do not attain a functional state in hippocampal circuits.

The functional alterations observed by us in new, adult-generated dentate gyrus neurons that are prematurely differentiated may find a structural correlate in the morphological alterations, most evident in 4-wk-old neurons identified after infection with a GFP-expressing retrovirus, i.e., reduced dendritic length and spine density.

We can assume that the birth datings of a neuron by BrdU and retroviral infection are comparable, and we consider that a neuron analyzed 4 wk post-infection is representative of the level of mid-late maturation, when the main neuronal structures are developed [19].

Altogether, it is plausible that the faster attainment of the terminally differentiated state, observed in adult-generated dentate gyrus neurons of mice with active transgene, may (following an initial faster development of the dendritic tree seen in neurons at 7 dpi) lead to a premature end of the developmental stages relative to the establishment and growth of the dendritic tree during the second and third week after birth. Only at later stages, i.e., in 10-wk-old neurons, emerges a tendency to recover, at least in part, normal values of spine density.

Neuronal structure and functional activity are correlated [71–75]. Indeed, the reduced dendritic structure affects spine formation (the latter being spatially constrained by dendrites) and ultimately the afferent synaptic input, since each spine receives one synaptic bouton [76]. As a result, the neuron has reduced capability to activate and integrate memory patterns, as our electrophysiological and behavioral data indicate.

### Continuous Requirement in Spatial Learning of Newly Generated Neurons

In conclusion, the selective anticipation of differentiation of new, adult-generated dentate gyrus neurons induced in the *PC3* transgenic mouse model reveals the critical role of developmental timing, even before terminal mitosis, for the generation of neurons that are fully able to integrate new spatial memories.

As our data indicate, the number of new neurons necessary to integrate new spatial information appears relatively small. In fact, the number of new dentate gyrus neurons generated during the shortest period of activation of the transgene, sufficient to prevent new spatial learning (about 20,000, i.e., 800–1,000 per day multiplied by 22 days elapsed before behavioral session 2), should not exceed 5%–6% of the total number of neurons in the dentate gyrus (less than one half million).

It is worth noting that the progenitor cells born before or during the first session of spatial training in Tg*PC3* OFF mice will normally differentiate without being affected by activation of the transgene during session 2, because in progenitor cells, the nestin promoter becomes physiologically inactive within 1 wk after their birth [7]. Thus, in Tg*PC3* OFF → ON mice, only the new neurons born in the 3–4-wk period of transgene activation preceding and during the second behavioral session appear to be responsible for the spatial learning and memory deficits observed.

Moreover, given that Tg*PC3* OFF → ON mice were significantly impaired in spatial tasks since the beginning of the second session, the premature differentiation of adult-generated hippocampal progenitor cells could prevent not only learning new spatial tasks but also the use of information acquired previously in normal condition, i.e., during session 1 before activation of the transgene.

Therefore, the picture emerging from our data is that the correct differentiation and integration into existing memory circuits of a relatively small population of new adult-generated neurons not more than 4 wk old are crucial not only for spatial learning but also, notably, for the use of memories consolidated in tasks previously performed. Conversely, neurons older than 4 wk appear less efficient. This unique role of younger adult-generated neurons in the maintenance of key memory functions may derive from their proposed ability to link temporally proximal events [23], and also entails the need for continuous renewal of new neurons as soon as they terminally differentiate. The late activation (i.e., c-*fos* expression) observed after training in 4–6-wk-old neurons by us and by the group of Frankland [21], might thus be related to subsequent steps of integration into spatial learning circuits.

The significant impairment of the hippocampal function observed in our model, i.e., the deficit of spatial learning in the Morris water and radial maze tests accompanied by deficit of dentate gyrus LTP, is not frequently observed in studies of spatial memory (e.g., see [77]).

It is plausible that the ablation of new neurons, while eliminating their functional contribution, may at least in some case trigger compensatory processes, e.g., increased proliferation of the surviving progenitors, or a plastic reassembly of the cytoarchitecture of circuits. In our model, the new neurons exist, albeit with a reduced functionality; thus, as our data strongly suggest, no compensatory processes are activated. Moreover, these altered new neurons appear to establish connections with the existing neurons and are thus integrated in previously-formed circuits. It might be the same as expanding a circuit by interspersing new elements with lower functionality. Therefore this model, assembling within spatial circuits new neurons generated in normal number but with altered maturation, can be considered the equivalent of an anatomical dominant-negative, and may give the advantage over ablative strategies of magnifying the ability of new neurons—variable depending on their maturation state—to integrate and/or recall traces of temporally proximal events from existing circuits.

## Materials and Methods

### Mouse lines and genotyping.

The bitransgenic *nestin*-rtTA/TRE-*PC3* mouse line is the progeny of two mouse lines, each carrying a transgene: *nestin*-rtTA transgene, encoding the tetracycline-regulated TransActivator driven by the rat *nestin* promoter, and TRE-*PC3* transgene, carrying the *PC3* coding region under control of the Tetracycline Responsive Elements. The nestin-rtTA and TRE-*PC3* transgenic mouse lines were generated previously [78,41], exploiting the tet-on system to control the activation of the *PC3* transgene [79], and they were maintained in heterozygosity in FVB background or in homozygosity in BDF1 (C57BL/6 x DBA/2) background, respectively. The bitransgenic *nestin*-rtTA/TRE-*PC3* mice used for experiments were isogenic, having been previously interbred for six or more generations. In bitransgenic mice, the TransActivator protein produced by the *nestin*-rtTA transgene binds and activates TRE-*PC3* in the presence of doxycycline, consequently inducing the expression of *PC3* transgene. Genotypization of bitransgenic *nestin*-rtTA/TRE-*PC3* mice was performed as described [41]. Animals were housed in standard breeding cages under a 12-h light-dark schedule at a constant temperature of 21 °C and underwent behavioral testing during the second half of the light period (between 2:00 and 5:00 p.m.) in sound-insulated rooms. All procedures involving mice were completed in accordance with the Istituto Superiore di Sanità (Italian Ministry of Health) and current European (directive 86/609/ECC) Ethical Committee guidelines.

### Animal treatment and sample preparation for immunohistochemistry.

Bitransgenic *nestin*-rtTA/TRE-*PC3* mice (named Tg*PC3* throughout this report) were treated for immunohistochemistry according to the following experimental protocol.

The *PC3* transgene was activated in Tg*PC3* mice P30 (termed Tg*PC3* ON mice) by doxycycline hydrochloride (2 mg/ml; MP Biomedicals) supplied to mice in drinking water containing 2.5% sucrose; at P90–94 mice received five daily bromodeoxyuridine (BrdU; 95 mg/kg i.p.) injections to detect dividing neurons. Immunohistochemical analyses were performed after BrdU treatment, at P95 and at P116. Tg*PC3* mice untreated with doxycycline (termed Tg*PC3* OFF mice) and WT mice (i.e., the progeny of crosses between *nestin*-rtTA and TRE-*PC3* transgenic mice, in which the TRE-*PC3* transgene was not inherited) were used as controls.

Brains were collected after transcardiac perfusion with 4% paraformaldehyde (PFA) in PBS–DEPC and kept overnight in PFA. Thereafter, brains were equilibrated in sucrose 30% and cryopreserved at −80 °C.

### Immunohistochemistry, BrdU labeling, immunofluorescence, and TUNEL analysis.

Immunohistochemistry was performed on serial sections cut transversely at 20-μm thickness at −25 °C in a cryostat from brains embedded in Tissue-Tek OCT (Sakura). Sections were then processed immunohistochemically for multiple labeling with BrdU and other cellular markers using fluorescent methods. BrdU incorporation was visualized by denaturing DNA through pre-treatment of sections with 2 N HCl 45 min at 37 °C followed by 0.1 M sodium borate buffer pH 8.5 for 10 min. Sections were then incubated with a rat monoclonal antibody against BrdU (Serotech; MCA2060; 1:150) together with other primary antibodies, as indicated: either mouse monoclonals raised against nestin (Chemicon International; MAB353; 1:150) and NeuN (Chemicon International; MAB377; 1:100), or rabbit polyclonal antibodies against Glial fibrillary acidic protein (*GFAP*; Promega Corporation; G560A; 1:150) and c-*fos* (Chemicon International; Ab-5 PC38T; 1:500), or goat polyclonal antibodies recognizing Doublecortin (DCX) (Santa Cruz Biotechnology; SC-8066; 1:200) or *NeuroD1* (R&D Systems; AF2746; 1:100). Another primary antibody used was a rabbit monoclonal antibody against Ki67 (LabVision Corporation; SP6; 1:100). These antigens were visualized with either TRITC (tetramethylrhodamine isothiocyanate)-conjugated donkey anti-rat (Jackson ImmunoResearch; BrdU), or TRITC-conjugated goat anti-rabbit (Sigma; Ki67), or Cy2-conjugated donkey anti-rabbit (Jackson ImmunoResearch; *GFAP*), or donkey anti-goat Cy2-conjugated (Jackson ImmunoResearch; DCX, *NeuroD1*) or also with donkey anti-mouse Alexa 647 (Invitrogen; nestin, NeuN, c-*fos*) secondary antibodies.

Terminal deoxynucleotidyl transferase-mediated biotinylated UTP nick end labeling (TUNEL) [80] was performed on cryostat sections using the in situ cell death detection kit (Roche Products), according to the instructions of the manufacturer. Apoptotic nuclei were visualized with 0.5% 3,3'-diaminobenzidine (DAB).

Images of the immunostained sections were obtained by laser scanning confocal microscopy using a TCS SP5 microscope (Leica Microsystem). All analyses were performed in sequential scanning mode to rule out cross-bleeding between channels.

### Quantification of cell number and volumes.

About 200 transverse sections spaced 20 μm apart and comprising the entire hippocampus were obtained from each brain; about one-in-ten series of sections (200 μm apart) were analyzed to count cells expressing the indicated marker throughout the whole rostro-caudal extent of the dentate gyrus. The total estimated number of cells within the dentate gyrus, positive for each of the indicated markers or combination of markers, was obtained by multiplying the average number of positive cells per section by the total number of 20-μm sections comprising the entire dentate gyrus [81,2,21]. At least three animals per group were analyzed. c-fos was analyzed in about one-in-ten series of 40-μm free-floating sections (400 μm apart).

Stereological analysis of volumes and of the absolute number of granule cells in the dentate gyrus was performed, analyzing every sixth section in a series of 40- μm coronal sections (thus spaced 240 μm). Total cell number was obtained according to the optical disector principle, by systematic sampling of unbiased counting frames of 15-μm side in each section. Nuclei considered in the count (identified by Hoechst 33258 staining) were those appearing throughout the different focal planes of each section, excluding those nuclei that intersected the exclusion boundaries of the counting frame, as defined by the optical disector principle [82]. To obtain the absolute cell number, the average cell number per disector volume (Nv; the disector volume being 15 × 15 × 40 μm^3^) was multiplied by the reference volume (i.e., the total volume of dentate gyrus; [82]). The reference volume was determined by summing the traced areas of dentate gyrus (or hippocampus) and multiplying this result by the distance between the sections analyzed (240 μm).

Measurements of positive cells and areas were obtained by computer-assisted analysis using the I.A.S. software (Delta Systems).

### Assay of β-galactosidase activity of transgenic mice.

To reveal the β-galactosidase activity of the β*-geo* reporter gene fused to the nestin-rtTA transgene [78], transverse sections from brains fixed as described above were post-fixed with 0.2% glutaraldehyde in PBS for 10 min, then washed three times for 5 min in *lacZ* wash buffer (1 M MgCl_2_, 1% sodium deoxycholate, 2% NP40 in PBS). Sections were then stained overnight at 30 °C with *lacZ* stain [*lacZ* wash buffer, 0.21% K_4_Fe(CN)_6_.3H_2_O, 0.16% K_3_Fe(CN)_6_, 25 mg/ml of 5-Bromo-4-Chloro-3-indolyl- β-D-galactopyranoside (X-Gal)], washed three times 5 min with PBS and mounted.

### Retrovirus-mediated labeling of new neurons in the mouse hippocampus.

The murine Moloney leukemia virus-based retroviral vector CAG-GFP [19] was used to infect only dividing cells at the moment of in vivo delivery. Retroviruses were propagated by transiently cotransfecting CAG-GFP with pHCMV-G vector (which expresses the VSV-G protein; [83]) in the packaging cell line Phoenix (human embryonic kidney cell line stably expressing the gag and pol proteins of Moloney murine leukemia virus; [84]). Cells at about 90% confluence in 90-mm dishes were transfected with 11.5 μg of CAG-GFP and with 13.5 μg of pHCMV-G, using calcium phosphate precipitation. Virus-containing supernatant was harvested 36, 48, and 60 h after the start of transfection. Frozen stocks were pooled and the virus was concentrated by centrifugation for 1.5 h in a Hitachi RPS40T rotor at 25,000 rpm. The concentrated virus solution (10^8^ pfu/ml) was infused (1.5 μl at 0.32 ml/min) by stereotaxic surgery into the right and left dentate gyrus of anesthetized P95 transgenic mice (anteroposterior = −2 mm from bregma; lateral = ±1.5 mm; ventral = 2 mm). The animal protocols were approved by the Istituto Superiore Sanità, Rome, Italy.

### Dendritic growth and spine density of GFP-positive neurons.

Dendritic analysis of GFP-positive neurons was performed by acquiring *z*-series of 15–25 optical sections at 1–1.5 μm of interval with a 40X oil lens, with the confocal system TCS SP5 (Leica Microsystem). Two-dimensional projections at maximum intensity of each *z*-series were generated with the LAS AF software platform (Leica Microsystem) in the TIFF format, and files were imported in the I.A.S. software (Delta Systems) to measure dendritic length. The number of branching points was counted manually in the same images. For each data point, 20–30 cells from two mice were analyzed (either control or with activated transgene). From the same GFP-positive neurons, the spines present on dendritic processes were imaged by acquiring *z*-series of 25–35 optical sections at 0.5 μm of interval with a 63X apochromatic oil lens, and a digital zoom of 3. The number of spines was counted manually on two-dimensional projections obtained by the LAS AF software. The linear spine density was then calculated by dividing the total number of spines by the length of the corresponding dendritic process.

### In vitro slice preparation and electrophysiology.

WT, Tg*PC3* OFF, and Tg*PC3* ON mice, 95 d old, were used for electrophysiological recordings. Tg*PC3* ON mice were used after animals were exposed for two months to doxycycline hydrochloride. Mice were deeply anesthetized with isofluorane inhalation and decapitated, and the brains were removed and immersed in cold “cutting” solution (4 °C) containing (in mM): 234 sucrose, 11 glucose, 24 NaHCO_3_, 2.5 KCl, 1.25 NaH_2_PO_4_, 10 MgSO_4_, and 0.5 CaCl_2_, gassed with 95% O_2_/5% CO_2_. Coronal slices (300 μm) were cut with a vibratome from a block of brain containing the dorsal hippocampus and then incubated in oxygenated artificial cerebrospinal fluid (aCSF) containing (in mM): 126 NaCl, 26 NaHCO_3_, 2.5 KCl, 1.25 NaH_2_PO_4_, 2 MgSO_4_, 2 CaCl_2_, and 10 glucose, gassed with 95% O_2_/5% CO_2_; pH 7.4, initially at 35 °C for 1 h, and subsequently at room temperature. Slices were then transferred to a submersed recording chamber and maintained at 32 ± 1 °C while being continuously perfused by fresh and oxygenated aCSF at a rate of ∼2–3 ml/min. Recordings were performed in the continuous presence of 100 μM picrotoxin to block inhibitory GABAergic transmission. Monopolar stimulating and recording electrodes consisted of glass pipettes (0.5–1 Mμ) filled with aCSF and placed in the middle of the outer molecular layer of the dentate gyrus or in the middle of the stratum radiatum. Field excitatory post-synaptic potentials (fEPSPs) were amplified using a Multiclamp 700B patch-clamp amplifier (Molecular Devices). A Digidata 1320 digitizer and PClamp9 (Molecular Devices) were used for data acquisition, analysis, and generation of stimuli. Input-output (I/O) curves were generated before inducing LTP. For I/O curves, stimulus intensities were adjusted to have afferent volleys of 50, 100, 150, 200 and 250 μV in all tested slices, and the resulting fEPSP slopes were calculated and averaged across 3–5 sweeps. For LTP experiments, fEPSPs were stimulated at 0.1 Hz. If a stable baseline of at least 10 min was achieved, LTP was induced by four trains of 100 stimuli at 100 Hz repeated every 20 s for recordings in the dentate gyrus and one train of 100 stimuli at 100 Hz in the CA1 area. Results are presented as means ± SEM. Data were averaged in 0.5-min bins.

### Behavioral tests.

All mice were tested in an open field and a plus maze [85,86] to assess locomotor abilities and anxiety levels. No statistically significant differences were found among the groups in both tasks (unpublished data).

The behavioral experiments were carried out in two phases, according to the schedule shown in Figure 3A. In the first phase, the following groups of mice, aged 3 mo, were used: Tg*PC3* ON (*n* = 10), in which the *PC3* transgene was activated at P30 by doxycycline administration; Tg*PC3* OFF (*n* = 8), in which doxycycline was not administered so that the *PC3* transgene was not activated; and WT (*n* = 12 treated with doxycycline; *n* = 11 untreated). At the end of the first phase, Tg*PC3* OFF (*n* = 8) and untreated control mice (WT, *n* = 11) were administered with doxycycline and, after day 22 of treatment, these two mice groups (labeled as Tg*PC3* OFF → ON and WT-doxy, respectively) were subjected to a second behavioral testing session.

The Morris water maze [54,55] was carried out in a circular swimming pool of 1.3 m in diameter, filled with opaque water at a temperature of 25 ± 1 °C and located in a room containing prominent extra-maze cues. A hidden, 15-cm diameter platform was used. In the first experimental phase, the training consisted of 18 trials (six trials per day, lasting a maximum of 60 s, with an intertrial interval of 30 min), with the platform left in the same position. After 3 d of learning, the platform was moved to the opposite position and reversal learning was monitored for two additional days. Probe tests (60 s) were carried out 24 h after both learning and reversal learning by removing the platform from the pool. In the second phase, Tg*PC3* OFF → ON and WT-doxy mice were submitted to a further session of spatial learning lasting 2 d, during which the platform was located in a different position from those used in the previous experimental phase. A probe test was carried out 24 h after learning. Behavior was evaluated by EthoVision software (Noldus Information Technology).

For the eight-arm radial maze [57,58], mice were singly housed, with water provided ad libitum, and gradually reduced to 85% of their free-feeding body weight. Throughout the experiment, mice were maintained at their reduced weight by being fed with a premeasured amount of food on each day. The maze apparatus was constructed of gray plastic maze, with eight identical arms radiating 37 cm from an octagonal starting platform (side 7 cm). On each training trial, a 20-mg food pellet was placed at the end of each arm (baits were not replaced) and the animal was placed on the central platform, facing a randomly selected direction. In both the first and second experimental phases, all groups of mice were submitted to one trial per day for 10 training days; each daily trial ended when eight choices were made or 15 min had elapsed. An arm choice was defined as placement of all paws on a maze arm. An error was noted when an animal entered a previously visited arm.

For the contextual and cued fear conditioning [87], the experiments were carried out in two different chambers. In both the first and second experimental phases, all mice were trained in conditioning chamber A (26 × 22 × 18 cm), made of transparent Plexiglas with a grid metal floor and located in a sound-insulated box lighted by a white tensor lamp (60 W). After an acclimatizing period lasting 120 s, a 30-s tone was administered (CS; 3 kHz, 80 dB). During the last 2 s of tone presentation, a foot-shock was delivered (US; 0.5 mA). Both CS and US ended simultaneously. Mice were left in the conditioning chamber for a further period of 30 s and then returned to their home cage. For the contextual conditioning test, 24 h after training mice were placed in the same chamber for 5 min. Two hours after the contextual test, mice were tested for cued conditioning in chamber B, made of black Plexiglas, with a floor of triangular shape, lighted by a blue tensor lamp (60 W) and perfumed by a vanilla essence diffuser. The test lasted 6 min, with CS administered during the last 3 min.

### Statistical analysis.

One-way ANOVA was used to analyze the c-*fos*-expressing neurons, the dendritic length, branching points, and spine density, as well as the levels of freezing in the contextual and cued fear conditioning and the electrophysiological data originating from slices from the different animal groups. Morris water maze and radial maze results were analyzed by two-way ANOVA. Individual between-group comparisons, where appropriate, were carried out by Fisher's PLSD post-hoc or Duncan multiple range test. Student's *t*-test was used to analyze the number of neurons and hippocampal volumes as well as data obtained in the same slices after LTP-inducing stimuli.

## Supporting Information

Figure S1 Types of Progenitor Cells Expressing β-Galactosidase Activity in Dentate Gyrus of Tg*PC3* ON Mice at P95 Shown are representative optical and confocal micrographs of the progenitor cell types expressing β-galactosidase, which, being encoded by the β*-geo* reporter gene fused to the nestin-rtTA transgene, matches the expression of the Tg*PC3* transgene.
(A) Progenitor cells expressing nestin (type-1 and type-2ab; brown) co-express β-galactosidase (labeled by X-gal staining, blue). Scale bar, 6 μm.
(B) A Ki67^+^/βGal^+^/GFAP^+^ cell (green, red, blue, respectively) corresponding to a type-1 cell, i.e., a putative stem cell with radial glial-like morphology having radial processes stained by GFAP and βGal, as indicated by two white arrowheads; in the left panel the three colors are merged.
(C) A Ki67^+^/βGal^+^/DCX^+^ progenitor cell (green, red, blue, respectively) corresponding to a type-2b cell (indicated by the arrow head), and (D) two Ki67^+^/βGal^–^/DCX^+^ cells, which are likely to correspond to type-3 cells (i.e., nestin^–^/DCX^+^; indicated by arrows); the Ki67^+^/βGal^+^/DCX^–^ cell on the left (indicated by an arrow head), presenting a morphology less elongated than type-1 cells, may belong to the type-2a. Scale bar, 7.5 μm. βGal^+^/NeuN^+^ cells, corresponding to stage 5–6 neurons, were not detected (unpublished data). The anti β-Galactosidase antibody used in (B–D) analyses was a mouse monoclonal (Cell Signaling Technology; 2372; 1:50). The primary antibody anti-nestin was visualized by horse anti-mouse biotinylated secondary antibody (Vector Laboratories; Kit ABC; 1:200).
(146 KB PDF)

Figure S2 Analysis of Progenitor Cell Types Expressing Ki67 in Dentate Gyrus (A) In P95 mice with the *PC3* transgene active since P30, no significant change was observed in the total number of proliferating Ki67-positive cells in the dentate gyrus, compared to control Tg*PC3* OFF mice.
However, the number of (B) proliferating progenitor cells type-1 (Ki67^+^/GFAP^+^/nestin^+^), (C) type-2ab (Ki67^+^/nestin^+^ and GFAP^–^) and (D) type-2b (Ki67^+^/nestin^+^/DCX^+^) decreased, whereas (E) proliferating type-3 progenitor cells increased significantly (Ki67^+^/DCX^+^ and nestin^–^). Such decrease of proliferating stem and progenitor cells with active transgene, i.e., nestin-positive cells, followed by increase of proliferating type-3 cells in which the expression of transgenic PC3 ceases, is compatible with a model where PC3 accelerates the transition to more mature progenitor cell types.
(F) Representative confocal images of progenitor cells in dentate gyrus of Tg*PC3* ON and OFF mice, either Ki67^+^/GFAP^+^/nestin^+^ (type-1, indicated by white arrows; green, blue, red, respectively, are merged), or Ki67^+^/nestin^+^ and GFAP^–^ (type-2ab, indicated by white arrowheads); scale bar, 50 μm. The cell numbers shown in (A–E) were measured as described in Materials and Methods and are represented as mean ± SEM. ** *p* < 0.01 versus Tg*PC3* OFF, Student's *t*-test; *n* = 4 for each group.
(2.97 MB PDF)

Figure S3 Stereological and Cell Survival Analyses in Tg*PC3* ON and OFF Mice In P95 mice having the *PC3* transgene active since P30, no difference was observed in comparison with control mice in (A) the volume of the dentate gyrus, (B) the whole hippocampus, (C) in the absolute number of neurons of dentate gyrus, or (D) in the number of neurons undergoing apoptosis (as detected by TUNEL). Stereological measurements were performed as described in Materials and Methods and are represented as mean ± SEM.
(E–I) Cell survival was analyzed also in the different subpopulations of progenitor cells and neurons in dentate gyrus, using caspase-3 as apoptotic marker. No change between Tg*PC3* ON and Tg*PC3* OFF mice was observed in the number of progenitor cells (E) type-1 and type-2a (caspase-3-nestin-positive and DCX-negative), (F) type-2b (caspase-3-nestin-DCX-positive), (G) type-3 (caspase-3-DCX-positive and nestin-negative), or of (H) differentiated neurons at stage 5 (caspase-3-DCX-NeuN-positive) or (I) differentiated neurons at stage 6 (caspase-3-NeuN-positive and DCX-negative). In (A–I) *p* > 0.05 versus Tg*PC3* OFF, Student's *t*-test; *n* = 3.
(J) Representative confocal images in dentate gyrus of Tg*PC3* ON and OFF mice of differentiated neurons at stage 5, either undergoing apoptosis (caspase-3-DCX-NeuN-positive, indicated by the white arrowhead; red, green, blue, respectively), or normal (DCX-NeuN-positive and caspase-3-negative); scale bar, 15 μm. The anti caspase-3 antibody used was a rabbit polyclonal (Cell Signaling Technology; 9661; 1:100).
(2.35 MB PDF)

Figure S4 Effects of the Activation of the *PC3* Transgene in Cells of the Subventricular Zone (A) In P95 mice with active *PC3* transgene, the number of new type B cells (BrdU/GFAP-positive) and (B) type C transit amplifying cells (BrdU/NG2-positive) did not change significantly, compared to control mice. The transgene was activated at P30, followed by five daily BrdU injections at P90, as in the experimental protocol of Figure 1.
(C) A significant decrease of about 40% was observed in type A neuroblast cells (BrdU/DCX-positive), known to derive from type C cells.
(D) Conversely, 1–5-d-old differentiated post-mitotic neurons in the SVZ (BrdU/NeuN-positive) increased more than 2-fold in mice with activated *PC3* transgene. As a whole, this suggests that *PC3* accelerates the transition of SVZ cells towards a differentiated state, as seen for dentate gyrus progenitor cells. The SVZ cell numbers are shown as mean ± SEM. ** *p* < 0.01 versus Tg*PC3* OFF, Student's *t*-test; *n* = 4 for each group. The anti NG2 antibody used was a rabbit polyclonal (Chemicon International; Ab5320; 1:100).
(225 KB PDF)

Figure S5 PC3 Transgene Activation Does Not Affect Nonlearning Aspects in the Morris Water Maze (A) Tg*PC3* ON (*n* = 10), Tg*PC3* OFF (*n* = 8) and WT (*n* = 23) mice showed no significant differences in the amount of time spent near walls (thigmotaxis) across learning (trial/block 1–18; *F*(2,38) = 3.19; *p* = not significant) and reversal learning (trial/block 19–30; *F*(2,38) = 3.03; *p* = not significant). Comparable levels of thigmotaxis were observed in Tg*PC3* OFF→ON (*n* = 8) and WT-doxy (*n* = 11) mice (*F*(1,17) = 3.47; *p* = not significant).
(B) Tg*PC3* ON (*n* = 10), Tg*PC3* OFF (*n* = 8) and WT (*n* = 23) mice showed no significant differences in swimming speed across learning (trial/block 1–18; *F*(2,38) = 2.18; *p* = not significant) and reversal learning (trial/block 19–30; *F*(2,38) = 1.99; *p* = not significant). Furthermore, no change in swimming speed was observed during the second experimental session, after *PC3* transgene activation in Tg*PC3* OFF → ON (*n* = 8) and WT-doxy (*n* = 11) (*F*(1,17) = 0.95; *p* = not significant).
(343 KB PDF)

Figure S6 Impaired Learning in the Morris Water Maze Is Not Related to Indirect Effects of *PC3* Over-Expression (A) Experimental timeline as a function of mouse age in days. To rule out the possibility of indirect effects of PC3 over-expression in nestin-positive cells on neighboring, mature granule cells, Tg*PC3* ON (*n* = 9) mice and their controls (WT, *n* = 8) were trained in the Morris water maze after withdrawal of doxycicline. Doxycicline treatment was suspended at P90, to allow 5 d of metabolization of the residual levels.
(B) The performance of Tg*PC*3 ON mice was significantly impaired during both learning (trial/block 1–18; *F*(1,15) = 12.84; *p* < 0.005) and reversal learning (trial/block 19–30; *F*(1,15) = 5.92; *p* < 0.05).
(C) Tg*PC3* ON spent a significantly smaller amount of time in the target quadrant both in probe 1 (*F*(1,15) = 9.93; *p* < 0.05) and probe 2 (*F*(1,15) = 7.09; *p* < 0.05). *p < 0.05 versus WT mice.
(410 KB PDF)

Figure S7 Lack of Integration of New Neurons into Dentate Gyrus Memory Circuits Correlates with Impairment of Spatial Learning in the Morris Water Maze (A) Escape latency during training (left panel) and time spent in quadrants during the probe test (right panel) of Tg*PC3* ON (*n* = 6) and WT (*n* = 6) mice analyzed for c-*fos* expression 4 wk after BrdU administration. Performance of Tg*PC3* ON mice was significantly impaired during both learning trials (*F*(1,10) = 22.99; *p* < 0.01) and probe test (*F*(1,10) = 6.86; *p* < 0.05).
(B) Escape latency during training (left panel) and time spent in quadrants during the probe test (right panel) of Tg*PC3* ON (*n* = 6) and WT (*n* = 6) mice analyzed for c-*fos* expression 6 wk after BrdU administration. As in the 4-wk group, performance of Tg*PC3* ON mice was significantly impaired during both learning trials (*F*(1,10) = 77.52; *p* < 0.001) and probe test (*F*(1,10) = 5.67; *p* < 0.05).*p < 0.05 in comparison with WT mice.
(426 KB PDF)

## Abbreviations

CA1 and CA3: areas of hippocampus
CS: conditioned stimulus
DCX: doublecortin
dpi: days post-infection
fEPSP: field excitatory postynaptic potential
GFAP: glial fibrillary acidic protein
HFS: high-frequency stimulation
LTP: long-term potentiation
SVZ: subventricular zone
Tg: transgene
US: unconditioned stimulus
WT: wild type
