# Supplementary material for: The Timing of Differentiation of Adult Hippocampal Neurons Is Crucial for Spatial Memory
Source: PLoS Biol. 2008 Oct 7;6(10):e246. doi: 10.1371/journal.pbio.0060246 (PMC2561078; doi:10.1371/journal.pbio.0060246)
Supplement: Supplementary file 1 [file pbio.0060246.sg001.pdf]

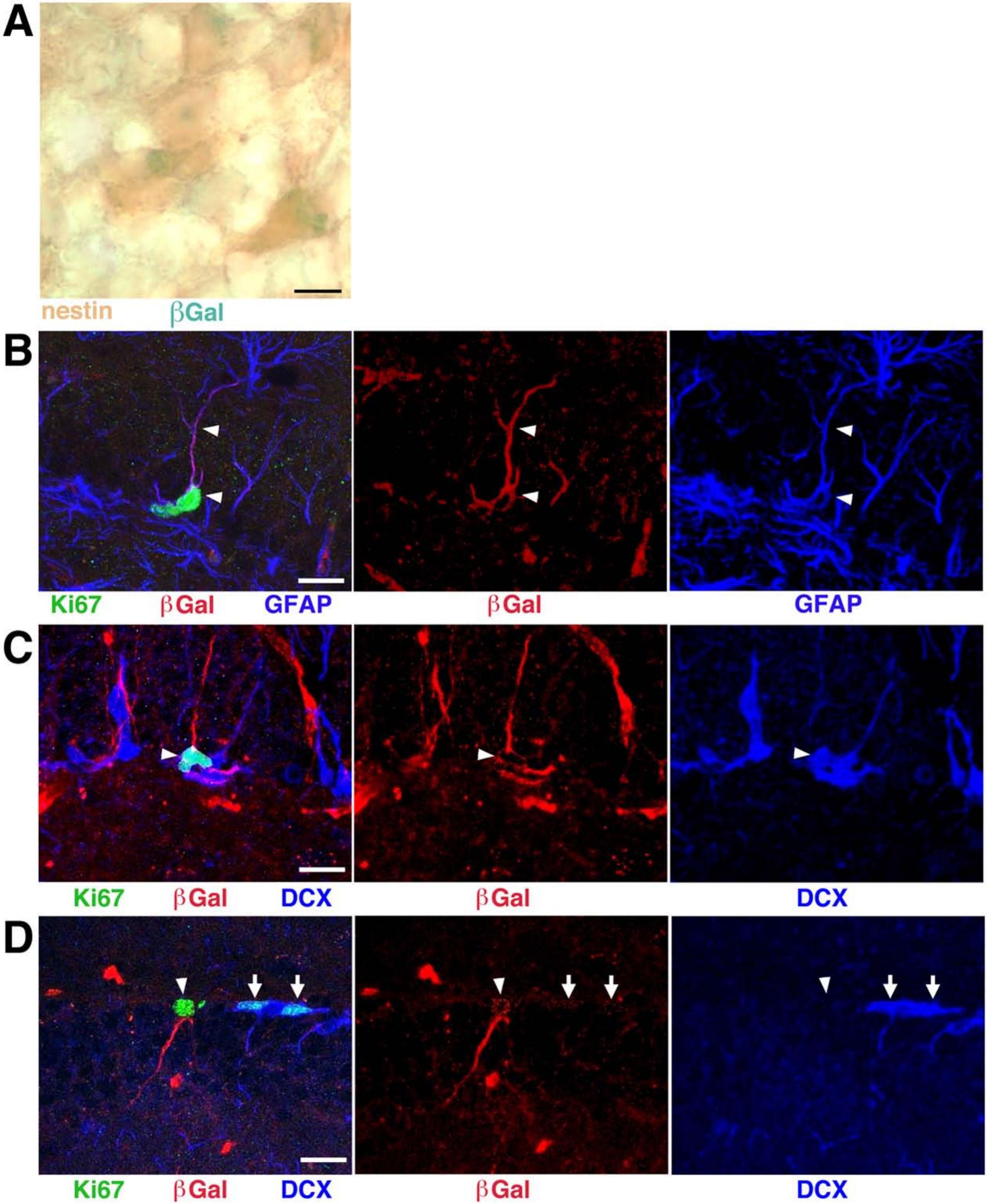
A
nestin
βGal
B
Ki67
βGal
GFAP
βGal
GFAP
C
Ki67
βGal
DCX
βGal
DCX
D
Ki67
βGal
DCX
βGal
DCX

Figure S1
