# Supplementary material for: The Timing of Differentiation of Adult Hippocampal Neurons Is Crucial for Spatial Memory
Source: PLoS Biol. 2008 Oct 7;6(10):e246. doi: 10.1371/journal.pbio.0060246 (PMC2561078; doi:10.1371/journal.pbio.0060246)
Supplement: Supplementary file 2 [file pbio.0060246.sg002.pdf]

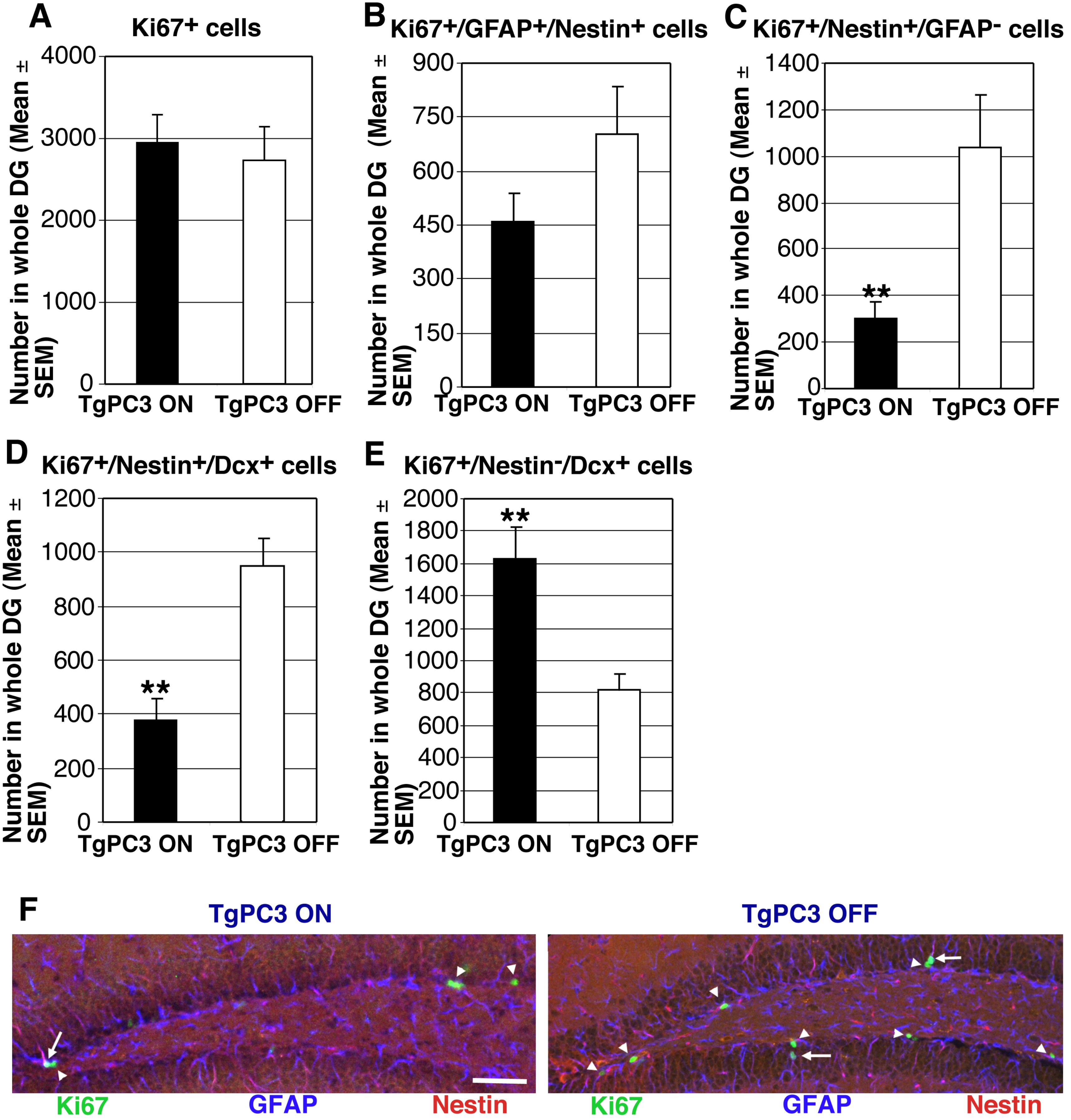

A
Ki67+ cells
Number in whole DG (Mean ± SEM)
4000
3000
2000
1000
0
TgPC3 ON
TgPC3 OFF
B
Ki67+/GFAP+/Nestin+ cells
Number in whole DG (Mean ± SEM)
900
750
600
450
300
150
0
TgPC3 ON
TgPC3 OFF
C
Ki67+/Nestin+/GFAP- cells
Number in whole DG (Mean ± SEM)
1400
1200
1000
800
600
400
200
0
**
TgPC3 ON
TgPC3 OFF
D
Ki67+/Nestin+/Dcx+ cells
Number in whole DG (Mean ± SEM)
1200
1000
800
600
400
200
0
**
TgPC3 ON
TgPC3 OFF
E
Ki67+/Nestin-/Dcx+ cells
Number in whole DG (Mean ± SEM)
2000
1800
1600
1400
1200
1000
800
600
400
200
0
**
TgPC3 ON
TgPC3 OFF
F
TgPC3 ON
TgPC3 OFF
Ki67
GFAP
Nestin
Ki67
GFAP
Nestin
