# Supplementary material for: The Timing of Differentiation of Adult Hippocampal Neurons Is Crucial for Spatial Memory
Source: PLoS Biol. 2008 Oct 7;6(10):e246. doi: 10.1371/journal.pbio.0060246 (PMC2561078; doi:10.1371/journal.pbio.0060246)
Supplement: Supplementary file 3 [file pbio.0060246.sg003.pdf]

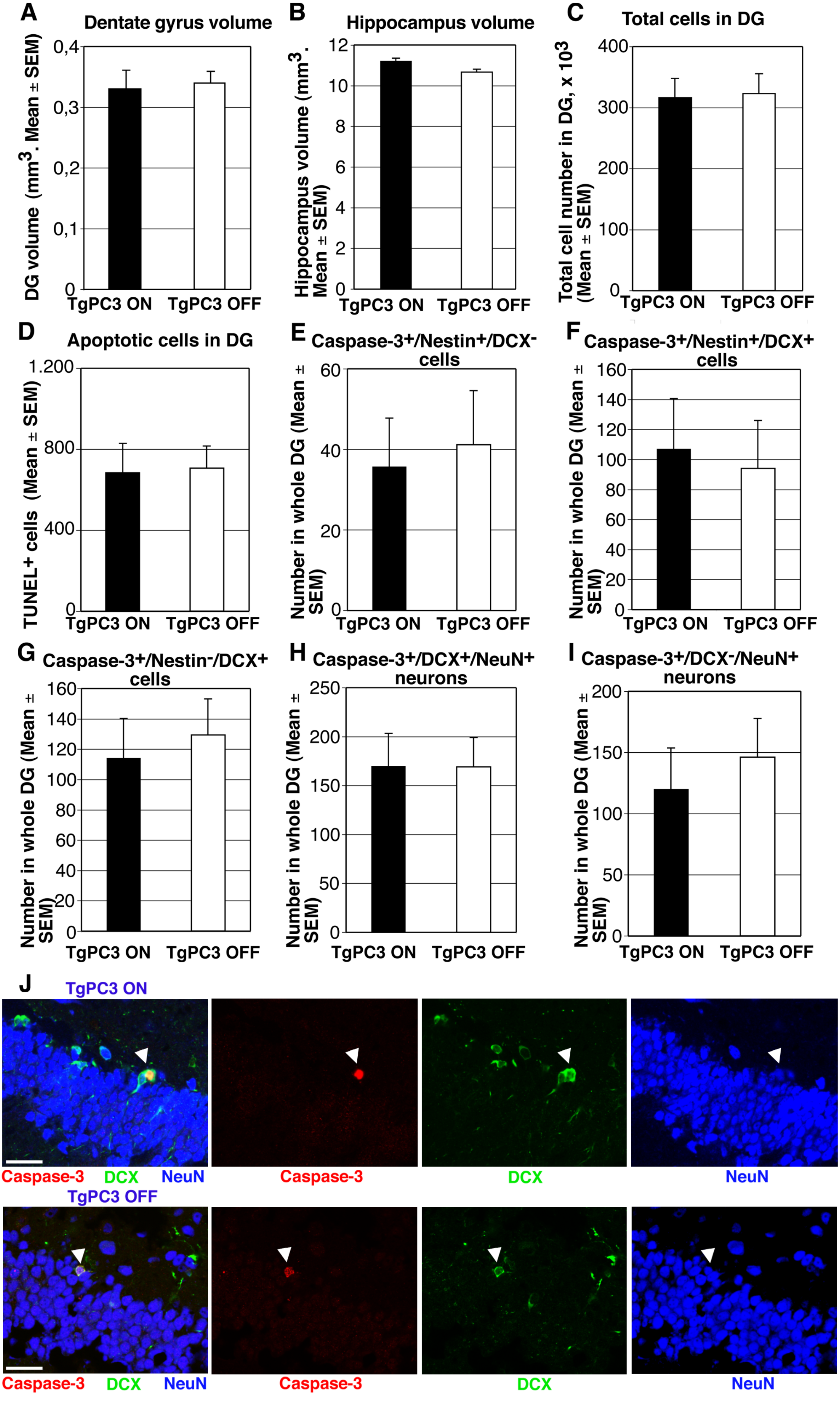
A
Dentate gyrus volume
DG volume (mm3. Mean ± SEM)
0,4
0,3
0,2
0,1
0
TgPC3 ON
TgPC3 OFF
B
Hippocampus volume
Hippocampus volume (mm3. Mean ± SEM)
12
10
8
6
4
2
0
TgPC3 ON
TgPC3 OFF
C
Total cells in DG
Total cell number in DG, x 103 (Mean ± SEM)
400
300
200
100
0
TgPC3 ON
TgPC3 OFF
D
Apoptotic cells in DG
TUNEL+ cells (Mean ± SEM)
1.200
800
400
0
TgPC3 ON
TgPC3 OFF
E
Caspase-3+/Nestin+/DCX- cells
Number in whole DG (Mean ± SEM)
60
40
20
0
TgPC3 ON
TgPC3 OFF
F
Caspase-3+/Nestin+/DCX+ cells
Number in whole DG (Mean ± SEM)
160
140
120
100
80
60
40
20
0
TgPC3 ON
TgPC3 OFF
G
Caspase-3+/Nestin-/DCX+ cells
Number in whole DG (Mean ± SEM)
160
140
120
100
80
60
40
20
0
TgPC3 ON
TgPC3 OFF
H
Caspase-3+/DCX+/NeuN+ neurons
Number in whole DG (Mean ± SEM)
250
200
150
100
50
0
TgPC3 ON
TgPC3 OFF
I
Caspase-3+/DCX-/NeuN+ neurons
Number in whole DG (Mean ± SEM)
200
150
100
50
0
TgPC3 ON
TgPC3 OFF
J
TgPC3 ON
Caspase-3 DCX NeuN
Caspase-3
DCX
NeuN
TgPC3 OFF
Caspase-3 DCX NeuN
Caspase-3
DCX
NeuN
