# Supplementary material for: The Timing of Differentiation of Adult Hippocampal Neurons Is Crucial for Spatial Memory
Source: PLoS Biol. 2008 Oct 7;6(10):e246. doi: 10.1371/journal.pbio.0060246 (PMC2561078; doi:10.1371/journal.pbio.0060246)
Supplement: Supplementary file 4 [file pbio.0060246.sg004.pdf]

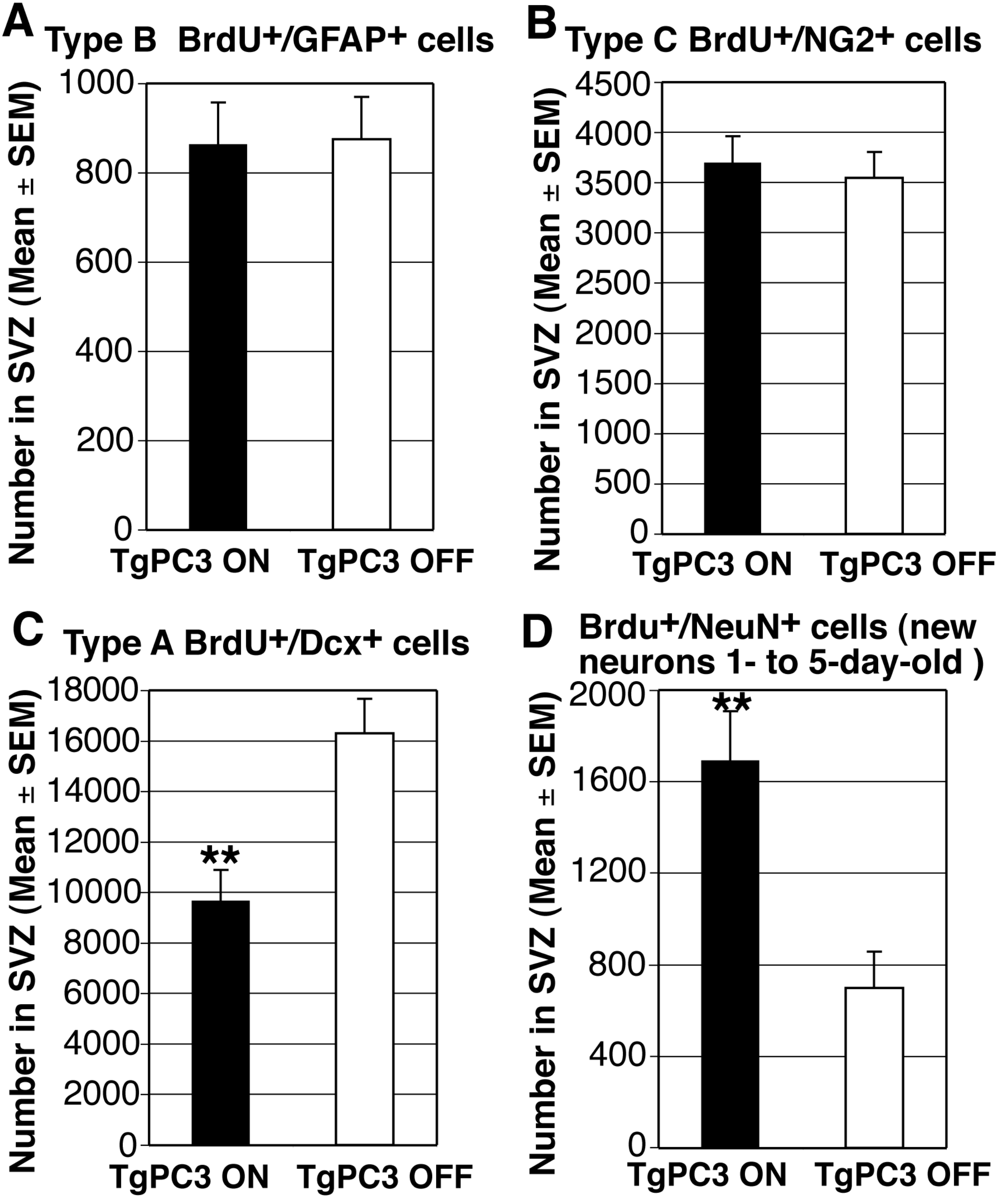
A
Type B BrdU+/GFAP+ cells
Number in SVZ (Mean ± SEM)
1000
800
600
400
200
0
TgPC3 ON
TgPC3 OFF
B
Type C BrdU+/NG2+ cells
Number in SVZ (Mean ± SEM)
4500
4000
3500
3000
2500
2000
1500
1000
500
0
TgPC3 ON
TgPC3 OFF
C
Type A BrdU+/Dcx+ cells
Number in SVZ (Mean ± SEM)
18000
16000
14000
12000
10000
8000
6000
4000
2000
0
**
TgPC3 ON
TgPC3 OFF
D
Brdu+/NeuN+ cells (new neurons 1- to 5-day-old)
Number in SVZ (Mean ± SEM)
2000
1600
1200
800
400
0
**
TgPC3 ON
TgPC3 OFF
