# Supplementary material for: The Timing of Differentiation of Adult Hippocampal Neurons Is Crucial for Spatial Memory
Source: PLoS Biol. 2008 Oct 7;6(10):e246. doi: 10.1371/journal.pbio.0060246 (PMC2561078; doi:10.1371/journal.pbio.0060246)
Supplement: Supplementary file 5 [file pbio.0060246.sg005.pdf]

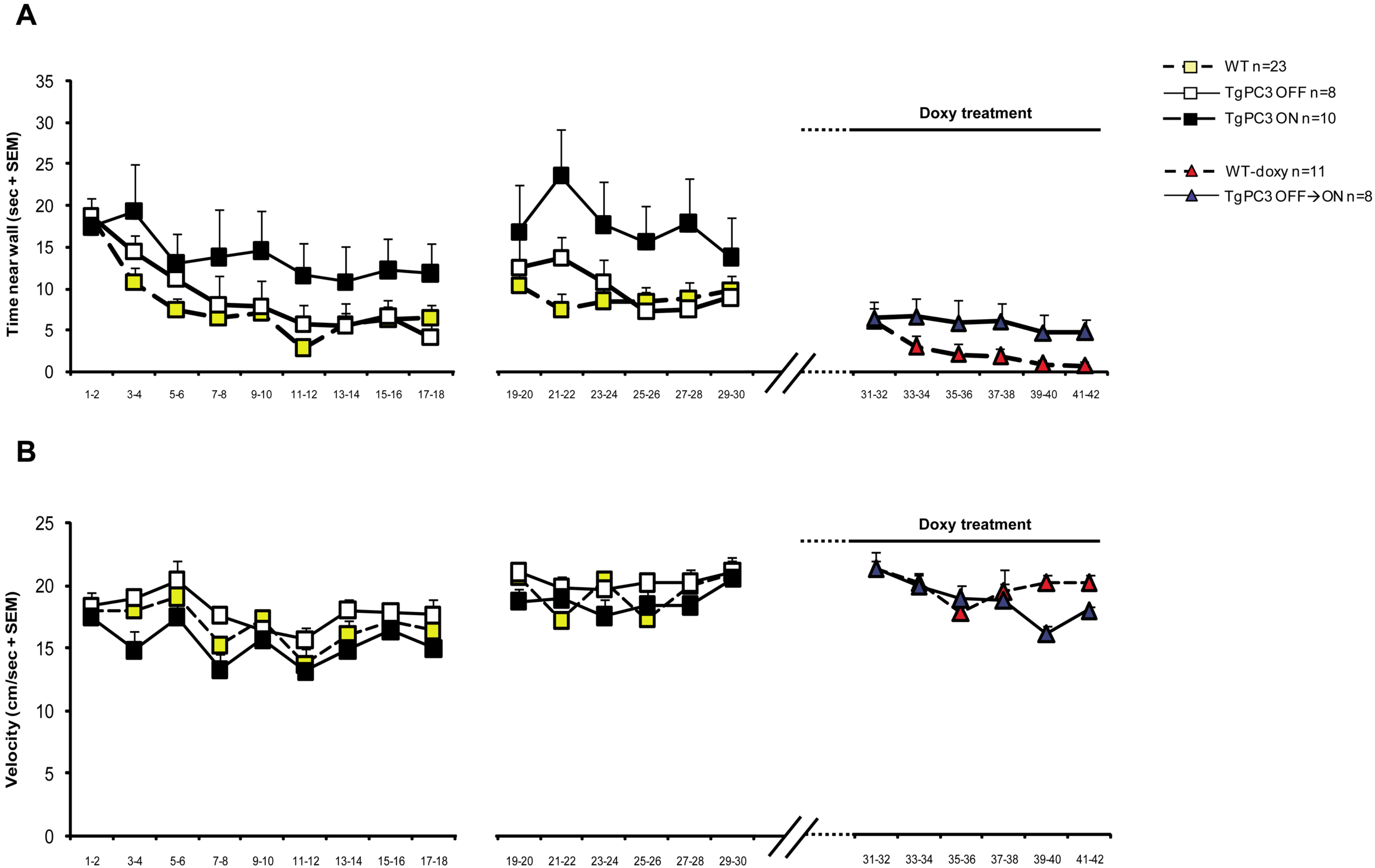

A
B
WT n=23
TgPC3 OFF n=8
TgPC3 ON n=10
WT-doxy n=11
TgPC3 OFF→ON n=8
Doxy treatment
Time near wall (sec + SEM)
Velocity (cm/sec + SEM)
1-2
3-4
5-6
7-8
9-10
11-12
13-14
15-16
17-18
19-20
21-22
23-24
25-26
27-28
29-30
31-32
33-34
35-36
37-38
39-40
41-42
