# Supplementary material for: The Timing of Differentiation of Adult Hippocampal Neurons Is Crucial for Spatial Memory
Source: PLoS Biol. 2008 Oct 7;6(10):e246. doi: 10.1371/journal.pbio.0060246 (PMC2561078; doi:10.1371/journal.pbio.0060246)
Supplement: Supplementary file 6 [file pbio.0060246.sg006.pdf]

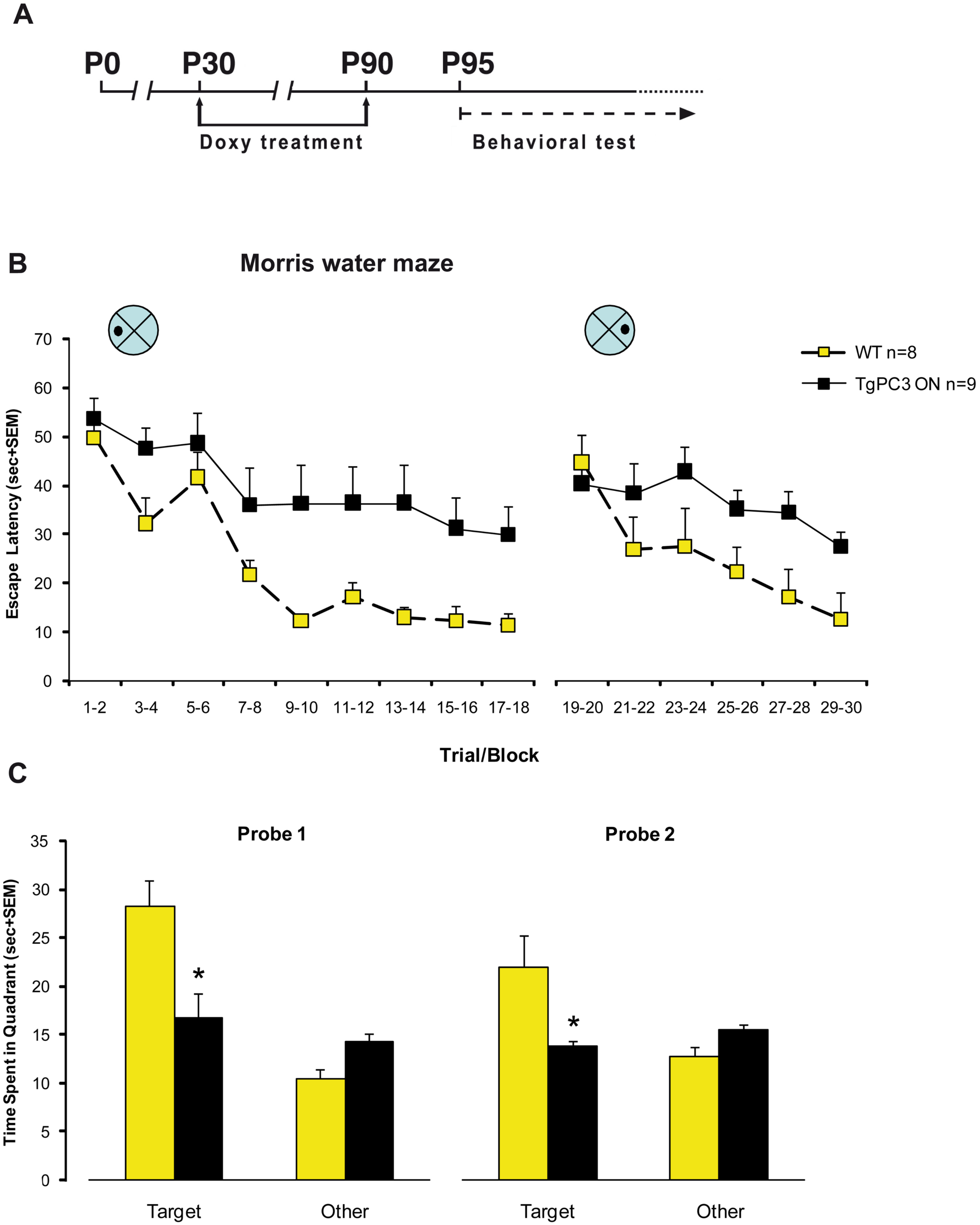
A
P0
P30
P90
P95
Doxy treatment
Behavioral test
B
Morris water maze
WT n=8
TgPC3 ON n=9
Escape Latency (sec+SEM)
70
60
50
40
30
20
10
0
1-2
3-4
5-6
7-8
9-10
11-12
13-14
15-16
17-18
19-20
21-22
23-24
25-26
27-28
29-30
Trial/Block
C
Probe 1
Probe 2
Time Spent in Quadrant (sec+SEM)
35
30
25
20
15
10
5
0
*
*
Target
Other
Target
Other
