# Supplementary material for: The Timing of Differentiation of Adult Hippocampal Neurons Is Crucial for Spatial Memory
Source: PLoS Biol. 2008 Oct 7;6(10):e246. doi: 10.1371/journal.pbio.0060246 (PMC2561078; doi:10.1371/journal.pbio.0060246)
Supplement: Supplementary file 7 [file pbio.0060246.sg007.pdf]

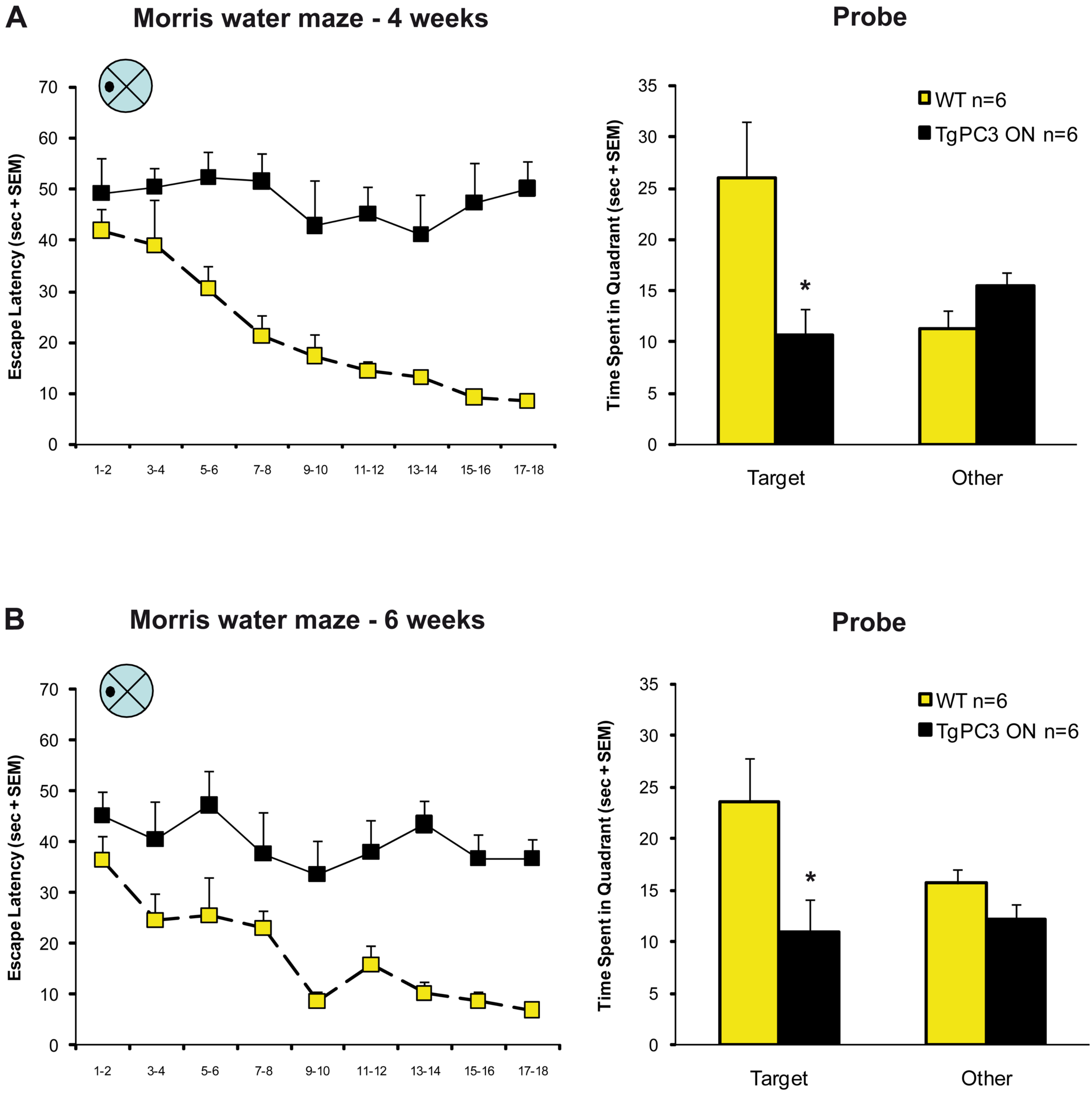
A
Morris water maze - 4 weeks
Escape Latency (sec + SEM)
0
10
20
30
40
50
60
70
1-2
3-4
5-6
7-8
9-10
11-12
13-14
15-16
17-18
Probe
Time Spent in Quadrant (sec + SEM)
0
5
10
15
20
25
30
35
WT n=6
TgPC3 ON n=6
*
Target
Other
B
Morris water maze - 6 weeks
Escape Latency (sec + SEM)
0
10
20
30
40
50
60
70
1-2
3-4
5-6
7-8
9-10
11-12
13-14
15-16
17-18
Probe
Time Spent in Quadrant (sec + SEM)
0
5
10
15
20
25
30
35
WT n=6
TgPC3 ON n=6
*
Target
Other
